# Integrating molecular markers into metabolic models improves genomic selection for Arabidopsis growth

**DOI:** 10.1038/s41467-020-16279-5

**Published:** 2020-05-15

**Authors:** Hao Tong, Anika Küken, Zoran Nikoloski

**Affiliations:** 1 Bioinformatics, Institute of Biochemistry and Biology, University of Potsdam, Potsdam, 14476 Germany; 2 Center of Plant Systems Biology and Biotechnology, Plovdiv, 4000 Bulgaria; 3 Systems Biology and Mathematical Modeling, Max Planck Institute of Molecular Plant Physiology, Potsdam, 14476 Germany

**Keywords:** Agricultural genetics, Data integration, Genetic markers, Secondary metabolism

## Abstract

The current trends of crop yield improvements are not expected to meet the projected rise in demand. Genomic selection uses molecular markers and machine learning to identify superior genotypes with improved traits, such as growth. Plant growth directly depends on rates of metabolic reactions which transform nutrients into the building blocks of biomass. Here, we predict growth of *Arabidopsis thaliana* accessions by employing genomic prediction of reaction rates estimated from accession-specific metabolic models. We demonstrate that, comparing to classical genomic selection on the available data sets for 67 accessions, our approach improves the prediction accuracy for growth within and across nitrogen environments by 32.6% and 51.4%, respectively, and from optimal nitrogen to low carbon environment by 50.4%. Therefore, integration of molecular markers into metabolic models offers an approach to predict traits directly related to metabolism, and its usefulness in breeding can be examined by gathering matching datasets in crops.

## Abstract (web summary)

An increase in genomic selection (GS) accuracy can accelerate genetic gain by shortening the breeding cycles. Here, the authors introduce a network-based GS method that uses metabolic models and improves the prediction accuracy of Arabidopsis growth within and across environments.

## Introduction

**Fig. 1:**
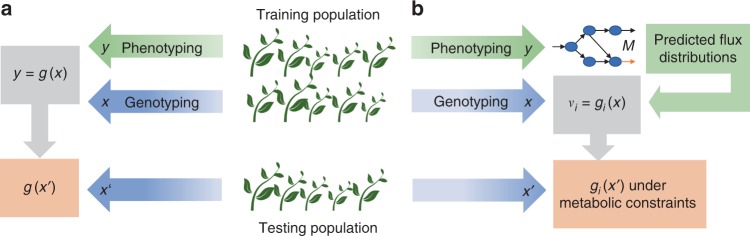
Comparison of classical and network-based genomic selection. **a** Classical genomic selection uses a statistical model $g\left( x \right)$, devised from genotypic data $x$ and phenotypic data $y$ in a training population, to predict the performance of individuals in a testing population with available genotypic data $x{\prime}$ only. **b** Network-based genomic selection uses phenotypic data to devise accession-specific metabolic models for the training population. The metabolic models are used to estimate steady-state fluxes for each metabolic reaction over the considered genotypes and to build respective statistical models $g_i\left( x \right)$ based on the genotyping data $x$. The statistical models are then used to identify a flux distribution, under metabolic constraints (e.g. steady-state), alongside the corresponding growth for a genotyped individual $x{\prime}$ from the testing population.

Advances in accuracy, precision, and throughput of molecular marker technologies have provided the basis for new approaches to improve agronomically important polygenic traits (e.g. fresh weight and yield)^1^. Genomic selection (GS) is currently considered the most promising breeding method to speed up the development and release of new genotypes^2^. It uses machine learning to integrate phenotypic data of a given trait with molecular markers (e.g. single nucleotide polymorphisms (SNPs)) in a statistical model for a training population. The model for the trait is then used to predict genomic estimated breeding values (GEBV) of genotypes in a testing population which have been genotyped but not phenotyped^3,4^ (Fig. 1a). The predicted GEBVs of unseen genotypes can be used for selection, even for complex traits with low heritability, without any further phenotyping. Therefore, an increase in GS accuracy can accelerate genetic gain by shortening the breeding cycles^2,5^. Yet, it remains elusive whether the accuracy of GS predictions within and, in particular, across environments can be improved^2,6,7^.

Although GS simultaneously estimates effects of markers by foregoing statistical testing, it does not integrate information of cellular networks available for model plants and some crops^8^. For instance, high-quality large-scale metabolic network models of *A. thaliana*, maize, and rice have been used to generate insights into genotype-phenotype relationships by using the constraint-based modeling framework that includes simplifying, but biochemically relevant constraints^9–11^. Metabolic network models include all known enzymatic functions of primary metabolism that influence growth. They further incorporate a biomass reaction that characterizes the chemical composition of a gram dry weight of the modeled plant or tissue in a specific environment^12^. Plant metabolic models have been employed to: (i) predict reaction rates through major pathways^13,14^, (ii) study the effect of manipulating pathways (e.g. photorespiration^15^ or introducing photorespiratory bypasses^16^), (iii) estimate the impact of nutrient deficiency on growth^11^, and (iv) compare the different types of photosynthesis^17,18^.

Here, we focus on plant growth as an agronomically relevant trait largely determined by the rate at which the available nutrients are transformed into the building blocks of biomass. We show that metabolic reaction rates (i.e. fluxes) are polygenic traits which can be predicted by GS and employed in estimating growth for a given genotype. As a result, we propose an approach for network-based GS (termed, netGS) that uses metabolic models and improves the prediction accuracy of classical GS for growth within and across environments (Fig. 1b). The formulation of netGS allows its applications for traits which are directly related to metabolism.

## Results

### Flux distribution of *A. thaliana* population

To integrate knowledge of metabolic network models in GS, we couple SNP data^19^ with predictions of steady-state fluxes from accession-specific metabolic models of 67 *A. thaliana* accessions with biomass reactions for optimal nitrogen (N) conditions (see Methods, Supplementary Fig. 1, Supplementary Data 1). The models are developed based on the data obtained from rosettes, where key processes relevant for growth take place. Estimating genome-wide steady-state fluxes with labeling approaches in a photoautotrophically grown *A. thaliana* rosette is currently practically infeasible^20^. To apply GS with fluxes as phenotypes, we first determine a reference steady-state flux distribution from *A. thaliana* accession Columbia (Col-0) (Supplementary Fig. 1). To this end, we use flux balance analysis (FBA)^21^ with a model that integrates a Col-0-specific biomass reaction and constraints on the rates of canonical pathways and key reactions (i.e. ratio of starch synthesis to sucrose synthesis rates and RuBisCO’s carboxylation to oxygenation rates) (Eq. 1) obtained from existing studies under optimal N^20^. This strategy has been used to accurately simulate the effects of photorespiratory bypasses^16^ and model different types of photosynthesis^18^. As a result, we predict that 336 of the 549 reactions (61.2%) in the metabolic model for Col-0 have non-zero fluxes (Supplementary Data 2). Most of the remaining zero-flux reactions are involved in export of amino acids to other tissues and in starch degradation (Supplementary Data 2). Since solutions obtained from FBA are often not unique, we also check the variability of the estimated reference flux distribution of Col-0. We show that the variability for more than 95% of reactions is negligible (see Methods, Supplementary Fig. 2, Supplementary Data 3) and, thus, does not affect the predictions that follow.

We obtain the flux distribution, under optimal N, for a model with a biomass reaction specific to another accession by minimizing the distance to the reference flux distribution of Col-0. To this end, we further impose an additional constraint that the ratio of predicted biomass fluxes, which model growth, fit the ratio of measured rosette fresh weights (Eq. 2, Fig. 1b, Supplementary Fig. 1, Supplementary Data 2). This method to estimate flux distributions is widely used in microbial and plant studies to estimate the flux distribution of mutant genotypes^15,22^. As a result, we obtain a flux profile for every reaction in the *A. thaliana* metabolic model over the population of 67 accessions grown under optimal N.

### Biological and statistical properties of flux distribution

**Fig. 2:**
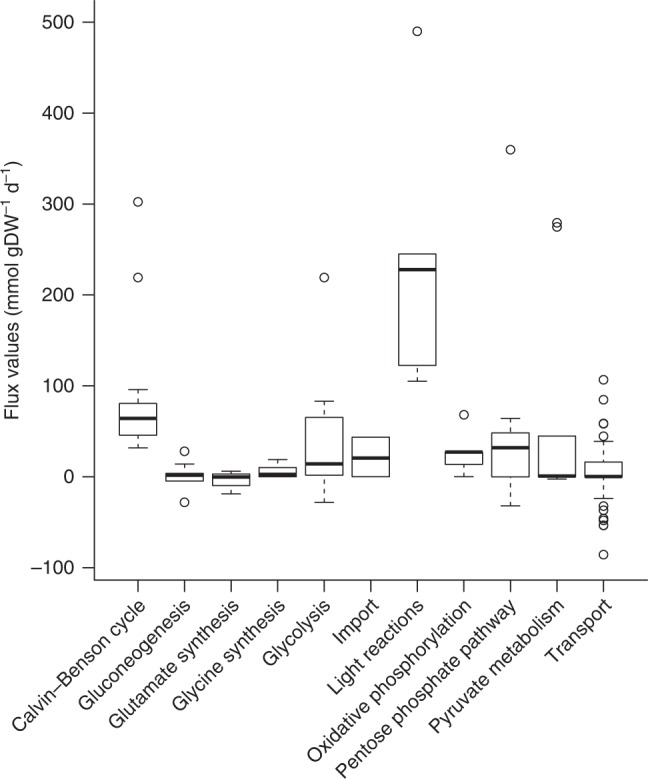
Flux ranges of metabolic systems in the metabolic model of *A. thaliana*. The metabolic systems with the most variable fluxes are shown in the x-axis. Largest variation is exhibited for glycolysis, photosynthesis light reactions, and pentose phosphate pathway. The remaining metabolic systems exhibit small fluxes, small variations, or both (Supplementary Fig. 3). *n*=3 to 76 fluxes in each metabolic system are used. Center line, median; box limits, 75th and 25th quartiles; whiskers, 1.5×interquartile range; points, outliers. Source data are provided as a Source Data file.

We next examine if the estimated flux distributions are biologically reasonable. Differences in fresh weight of 67 *A. thaliana* accessions are expected to be directly linked to alterations in nutrient acquisition, fixation, and (re)allocation as well energy demand between accessions. Indeed, we find that the largest flux ranges across the 67 accessions are observed for reactions involved in: photosynthesis, i.e. the Calvin–Benson cycle and light reactions, glycolysis, oxidative phosphorylation, pentose phosphate pathway, gluconeogenesis, glutamate synthesis and degradation, glycine synthesis, and pyruvate metabolism (Fig. 2, Supplementary Fig. 3). More specifically, we find that the average ratio between the RuBisCO’s carboxylation and oxygenation rates exhibits a 41.7% decrease, while the average ratio the between starch synthesis and synthesis rates shows a 59.5% decrease relative to the measured value in Col-0, demonstrating an expected variability in the flux through canonical pathways to explain differences in fresh weight^23^ (Supplementary Table 1).

To further demonstrate that the predicted fluxes are biochemically feasible, we contrast the predictions with measurements of maximal rate (*V*_max_) for six enzymes: nitrate reductase, glucose-1-phosphate adenylyltransferase, glutamine synthase, malate dehydrogenase, glutamate synthase, and fumarate hydratase^24^. We expect that if the predicted fluxes are biochemically feasible, they must not exceed the accession-specific *V*_max_. Indeed, we find that the predicted fluxes in every accession are in line with this expectation for five out of the six enzymes, and there are only very small deviations in nitrate reductase for 30 accessions (Supplementary Data 4). Therefore, the estimated fluxes are biologically feasible and can be used in further analyses.

We next quantify the similarity between every pair of accessions based on the Pearson correlation of the accession-specific data on SNPs, measured metabolite levels, and estimated fluxes, and investigate the congruence of the resulting matrices by the Mantel correlation^24^ (Supplementary Fig. 4). To this end, we use two types of SNP data: SNPs which fall in coding sequences of genes included in the metabolic models, termed enzymatic SNPs (1824), and all SNPs, termed genome-wide SNPs (180,859) (see Methods). The matrices capturing the similarity of accessions based on the enzymatic and genome-wide SNPs exhibit a significant and large Mantel correlation (0.94, *p-*value < 10^−30^), while all other pairs show negligible correlations (Supplementary Table 2). These findings show that enzymatic SNPs are representative markers, and that relationships between accessions based on genomic data are not congruent with those based on metabolic phenotypes. The latter is consistent with established characterizations of metabolic phenotypes as polygenic traits of relatively low heritability^25^. As a result, we focus the analysis on enzymatic SNPs, and show that the findings also hold with the larger set of genome-wide SNPs.

The flux distribution of every non-reference accession is obtained independently of data and models of other non-reference accessions. So we next investigate if the predicted fluxes are suitable for statistical modeling. We observe that 293 reactions (i.e. 87.2% of reactions with non-zero flux) show coefficients of variation greater than 10% (Supplementary Fig. 5, Supplementary Data 5), a level of variation that is sufficient for statistical modeling. If every flux exhibits high correlation to measured fresh weight, we would not be able to improve GS accuracy by integrating marker data into metabolic models. We find that 96 reactions (28.6%) show an absolute value of the Pearson correlation coefficient smaller than 0.9, with the smallest of these corresponding to the flux of water diffusion between different cellular compartments (Supplementary Data 6). Therefore, not all fluxes mimic the fresh weight data, used as constraints in the flux estimation, and they may be used to improve the accuracy of genomic prediction of growth.

### Improvement of GS accuracy by usage of metabolic models

We next employ the enzymatic SNPs and devise a statistical model for the flux of each reaction. To this end, we opt to use a state-of-the-art statistical approach for GS, the ridge regression best linear unbiased prediction (rrBLUP) with 3-fold cross-validation^26^. We employ the resulting models to determine flux GEBVs for each reaction (Supplementary Fig. 1). The average accuracy of the flux models is 0.225, which is lower than the accuracy of predicted growth (0.241, biomass reaction) (Supplementary Data 6). The prediction accuracy for fluxes of only 95 reactions (28.3%) is higher than that of growth, and it is negative for five fluxes (1.5%) (Supplementary Fig. 6A). Moreover, consistent with the high correlations between estimated fluxes and measured biomass, the accuracy of models for 223 reactions (66.4%) falls in a narrow range (i.e. between 0.22 and 0.26) to that of growth. However, the fluxes with most accurate models (larger than 0.40) exhibit lower correlations to biomass (~0.72) (Supplementary Data 6). Similar findings hold for genome-wide SNPs, where the prediction accuracy for fluxes ranges between −0.062 and 0.464, with an average of 0.339 (Supplementary Fig. 6B, Supplementary Data 6).

**Fig. 3:**
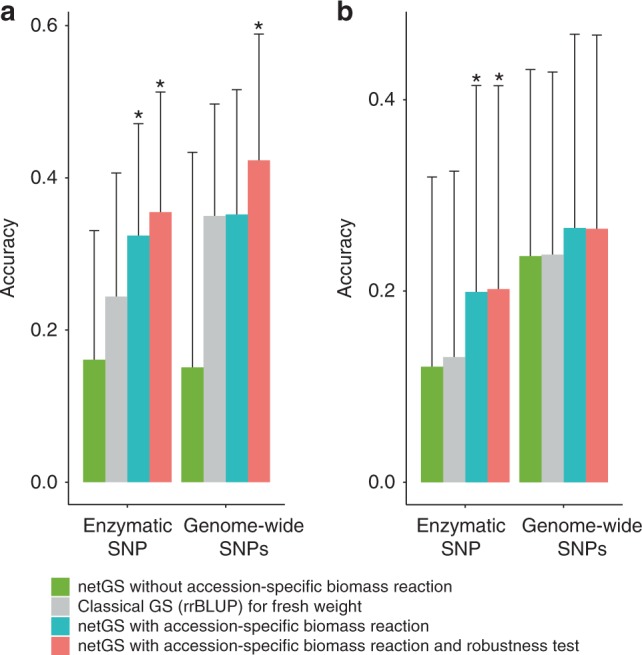
Comparison of GS prediction accuracies for growth. The predictions for growth are performed based on the classical GS (rrBLUP approach, gray), netGS using biomass reaction specific to Col-0 only (green), netGS using accession-specific biomass reaction (blue), and netGS with additional robustness analysis (red). The prediction accuracies are measured by the mean values of Pearson correlation coefficient between measure and predicted biomass using either enzymatic SNPs or genome-wide SNPs with 150 cross-validations (i.e. 50 repetitions of 3-fold cross-validation). The comparison is presented for two scenarios: (**a**) the optimal N condition and (**b**) the low N condition using metabolic models based on data from optimal N condition. The prediction accuracies of netGS with accession-specific biomass reaction is significantly higher than the classical GS using enzymatic SNPs (*p-*value = 6.28 × 10^−6^ within optimal N condition and *p-*value = 2.72 × 10^−6^ from optimal N to low N condition, two-sided paired t-test, *n* = 150 cross-validations, *denotes significant at level 0.01). Data are presented as mean values and standard deviation (s.d.). Source data are provided as a Source Data file.

However, estimations of GEBV for fluxes as traits cannot be directly used in netGS for growth, since the predictions resulting from statistical models may not respect the basic physico-chemical constraints (e.g. mass balance and upper bounds on fluxes). To address this problem, we determine the closest steady-state flux distribution to the predicted flux GEBVs which exceed the GEBVs of the flux through the biomass reaction (Eq. 4, Supplementary Fig. 1). The resulting steady-state flux GEBVs are associated with a flux through the biomass reaction which we use as a GEBV for growth. The average accuracy of netGS from a 3-fold cross-validation is 0.31, which leads to a significant increase of 28.2% over the accuracy for prediction of fresh weight (as a proxy for growth) using the classical GS (Supplementary Table 3, *p-*value = 9.01×10^−5^, paired t-test). We also investigate the effect of using only the flux GEBVs from statistical models with accuracy above a given threshold. Following this strategy, we demonstrate that the accuracy of netGS can be further increased to 32.6% relative to the classical GS when using only flux models with prediction accuracy larger than that for fresh weight (Fig. 3a, Supplementary Table 3, *p-*value = 6.28×10^−6^, paired t-test).

It has been shown that larger differences between the training and testing populations lead to worse GS accuracy^27^. However, this remarkably does not hold for netGS: in the cross-validation case with the largest population difference, with a coancestry coefficient^28^ of 0.21, we find that the accuracy of the classical GS is nearly zero, but that of netGS reaches 0.31 (Supplementary Data 7). However, while netGS cannot further improve 83.3% of the cases with accuracies larger than 0.4 from the classical GS, and we find that all cases with negative prediction accuracy from the classical GS are improved by netGS (Supplementary Fig. 7, Supplementary Data 7).

Since netGS combines the interdependence of fluxes with measured metabolite levels to create accession-specific models, we also examine the effect of integrating accession-specific metabolite levels. To this end, we repeat the calculations by only using the biomass reaction for Col-0 across all accessions. Our findings show that the prediction accuracy of netGS without accession-specific biomass reactions is decreased by ~50% (Fig. 3a, Supplementary Data 7). Therefore, the improved performance of netGS can be attributed to combining network information with data on accession-specific metabolite levels. We also examine if the prediction accuracy of netGS is affected by alteration of the reference flux distribution. To this end, we performed a robustness analysis, and show that for 50 reference distributions close to that of Col-0, netGS further improves the prediction accuracy relative to the classical GS with a significant increase of 45.5% (see Methods, Fig. 3a, Supplementary Data 8, *p-*value = 1.04×10^−9^, paired t-test). Altogether, netGS achieves better predictions within the same environment than the classical GS when using enzymatic SNPs and similar performance when using genome-wide SNPs, and the robustness analysis indicates further improvements (Fig. 3a, Supplementary Data 7, Supplementary Data 8).

### netGS improves prediction for unseen environment

Successful deployment of GS in plant breeding depends on the availability of data for phenotypes in possible future environments^1^. As a result, we next ask if netGS can be used to make predictions for an unseen environment for which accession-specific models are not available. To this end, we take advantage of the fact that difference in environments is principally reflected in the exchange (i.e. import and export) fluxes on the boundary of the metabolic network. The alterations in the exchange fluxes then propagate to and affect the rest of the fluxes in the network. To determine these alterations, we use a steady-state flux distribution of the reference genome, in this case Col-0, in a second environment to determine the ratio of exchange fluxes between the two environments. To arrive at the reference flux distribution for Col-0 in the second environment, we identify the closest flux distribution compatible with the change in biomass between the two environments (Eq. 5).

To test the approach, we employ the flux distribution for Col-0 under optimal N together with a biomass reaction specific to low N, to determine the respective reference flux distribution for low N condition. The fluxes of all exchange reactions are smaller under low N compared to optimal N, with an average fold-change of 0.71, reflecting the smaller fresh weight under low N (Supplementary Table 4). We find that the flux value of nitrate import reaction in low N condition is, as expected, smaller than in optimal N condition, with a fold-change of 0.77. Further, the flux of nitrate import under low N falls in the range of all observed accessions under optimal N (i.e. between 3.08 and 8.46 mmol g DW^−1^ d^−1^) (Supplementary Table 4). The import of phosphate changes the most between the two conditions, with a fold-change of 0.52, while the import of photons changes the least, with a fold-change of 0.98 (Supplementary Table 4).

With the reference flux distribution under low N established, we next seek to determine the steady-state flux distribution for an accession under low N. To this end, we find the closest steady-state flux distribution to the flux GEBV from optimal N that is compatible with constraints on the exchange fluxes under low N, together with an accession-specific biomass reaction under optimal N (Eq. 6). Therefore, models for low N are not used for any accessions other than the reference. We then use the resulting flux distribution to determine GEBV for growth under low N. Following this approach, we find that the across-environment prediction accuracy of netGS is similar to that of the classical GS when considering only the constraint on nitrate import. However, when we additionally impose constraints on import of photons, carbon dioxide, and water, we show that netGS using enzymatic SNPs improves the across-environment prediction accuracy by 51.4% relative to the classical GS (Fig. 3b, Supplementary Data 9, *p-*value = 2.72×10^−6^, paired t-test). Similar trends are observed with genome-wide SNPs (Fig. 3b, Supplementary Data 9).

### Effects of population structure and statistical approach

The power of GS may depend on the statistical approach used and can be affected by the population structure^29^. To determine the effect of other statistical approaches, we use BayesC with enzymatic SNPs. We observe that the prediction accuracy of GS with BayesC is similar to that based on rrBLUP (Supplementary Data 10, Supplementary Data 11). We also find that netGS with BayesC to determine models for reaction fluxes improves the within-environment prediction accuracy by 31.6% (Supplementary Data 10, *p-*value = 1.21×10^−5^, paired t-test), while the across-environment prediction accuracy is improved by 67.8% in comparison to the classical GS (Supplementary Data 11, *p-*value = 7.13×10^−9^, paired t-test). To examine the effects of the population structure, we employ the first ten principal components (PCs) with the enzymatic as well as genome-wide SNP. We find that the inclusion of the first ten PCs improves the prediction based on the enzymatic SNPs for both GS and netGS within and across environments using rrBLUP, respectively; however, the effects with the genome-wide SNPs are negligible. In all examined cases with the different statistical approaches (i.e. rrBLUP and BayesC), netGS consistently outperforms the classical GS (Supplementary Data 12, Supplementary Data 13). Therefore, netGS offers a cost-effective alternative to consideration of environmental effects in GS for metabolic traits as it does not rely on data from large-scale phenotyping under multiple environments.

### Effects of constraint-based approach for flux estimation

The flux distribution of the reference and the other genotypes in netGS are determined based on FBA in addition with constraints that render a robust and biologically meaningful flux distribution. Nevertheless, there are other approaches that can be used to estimate fluxes, including the parsimonious FBA (pFBA)^30^. By using pFBA, we find that the prediction accuracy of netGS within and across nitrogen environments are up to 39.4% (Supplementary Data 14, *p-*value = 2.36×10^−7^, paired t-test) and 19.6% (Supplementary Data 15, *p-*value = 0.02, paired t-test) higher than those of classical GS. Therefore, the findings between pFBA and FBA are qualitatively similar.

### Application of netGS with other environments

The formulation of netGS allows transferability of the statistical models for metabolic fluxes between two environments as long as there are estimates for the flux distributions of the reference genotype for the respective environments. While we showed that netGS improves prediction accuracy between environments which differ in the same factor, namely, availability of nitrogen, it remains questionable if netGS leads to increase in performance if models are transferred between two environments which show differences in two factors, namely nitrogen and carbon. To test the applicability of netGS in such a scenario, we applied our netGS approach estimating from optimal nitrogen condition to a low carbon condition. By using a constraint on the ratio of carbon dioxide import flux and statistical models for the fluxes from optimal nitrogen condition, we show that netGS lead to an improvement up to 50.4% comparing with the classical GS (Supplementary Data 16, *p-*value = 2.29×10^−6^, paired t-test).

## Discussion

We demonstrate that netGS provides the means to integrate molecular markers in large-scale metabolic models, which on the data set from 67 *A. thaliana* accessions resulted in improved prediction of plant growth using enzymatic and genome-wide SNPs. Plant performance has classically been predicted by crop growth models (CGMs), which cast agronomically relevant traits, such as yield and growth, as a function of other morphological and physiological traits as well as environmental variables^31^. Two approaches already allow for integration of genome-wide SNPs in CGMs by simultaneously inferring the physiological model parameters and effects of genome-wide SNPs on the parameters in a Bayesian framework^32,33^. While these approaches have been shown to improve the accuracy of predictions over the classical GS and facilitate the modeling and inference of genotype-by-environment interactions, they use information about the ranges of the physiological parameters over all genotypes and require environmental variables as input. In addition, CGM approaches are statistical in nature and do not provide mechanistic insights about the reasons for the particular performance of specified genotypes. In contrast, netGS does not directly include environmental variables; the environment is reflected in the metabolomics data which are used in the development of the environment-specific models for the reference genotype. Furthermore, by using metabolic network models and the flux distributions, as an intermediate phenotype, netGS provides mechanistic understanding for the differences in performance between two genotypes.

Our study provides a proof-of-principle that netGS provides a feasible approach to predict growth for the model plant *A. thaliana*. The approach is tested by using accession-specific models which integrate metabolomics data from *A. thaliana* rosettes in respective condition- and accession-specific biomass reactions for the relatively small population. Future simulation studies will examine how changes in the size of the used population may affect the prediction accuracies, which in the examined datasets show comparable uncertainties with those resulting from the classical GS.

As a constraint-based modeling approach, netGS can be extended to integrate transcriptomics, proteomics and metabolomics data^34–37^ and, thereby, impose additional constraints and explore their effect on prediction accuracies, as done in classical prediction of fresh weight^38^. The current state of plant metabolic modeling does not yet allow incorporation of information about catalytic rates, as plant-specific information about this is still lacking. Future studies may aim to expand netGS to integrated models which consider multiple, interconnected metabolic networks of different tissues^39,40^.

Our findings suggest that the improved prediction accuracies within and across environments may be due to the consideration of accession-specific metabolic networks and flux phenotypes that tacitly include interactions between molecular markers which are otherwise challenging to integrate in statistical models. Most importantly, our results from the studied *A. thaliana* population show that environment-specific metabolic models are needed only for the reference genotype to facilitate improved accuracy of prediction across environments. To further decrease the effort for generating genotype-specific metabolic models for a single environment, necessary in netGS, future studies may investigate the generation of biomass reactions directly from SNPs data following the classical GS.

Due to the constraint-based formulation, netGS appears facile to apply for traits directly related to metabolism in agronomically relevant crops. However, testing the performance of netGS on metabolic traits in crops, e.g. maize and rice, that have experienced recent history of intense selection will necessitate the assembly of high-quality metabolic models. Another difficulty is that the models must be able to reproduce growth and other metabolic traits of representative organs or the plant as a whole, before they can be employed for the estimation of fluxes, as intermediate traits. In addition, dedicated experiments will have to be designed to assemble accession-specific biomass reactions which are able to simulate metabolic traits of interest in specific contexts. Therefore, the usage of netGS in decreasing the phenotyping effort and expediting the development of superior crop genotypes remains to be validated in future studies that address the aforementioned challenges.

## Methods

### Plant materials and datasets

We used data gathered from a panel of 97 diverse *A. thaliana* accessions in a previous study^41^. For two nitrogen (N) conditions (optimal N and low N), all accessions were grown under 12-h photoperiod; for low carbon (C) condition, all accessions were grown under 8-h photoperiod^42^. In the low N condition, the soil was constituted of 50% (v/v) white peat (Gramoflor GmbH) and 30% (v/v) fine and 20% (v/v) coarse-grained vermiculite (AGRA-RHP, Kausek GmbH). Additionally, 260 mg K_2_HPO_4_, 396 mg GRANUKAL 85 (80% [w/v] CaCO_3_ and 5% [w/v] MgCO_3_, Kreidewerke Dammann KG), 1.6 mg Fetrilon-Combi micronutrient fertilizer (BASF AG), and 30 mL of tap water was added per 100-mL pot. In the optimal N condition, a supplement of 90 mg solid NH_4_NO_3_ was added to low N soil per 100-mL pot. The inorganic N per pot in low N and optimal N was ca. 1.25 and 31.5 mg, respectively^43^. For the low C condition, the soil substrate was GS90 (peat, clay, coconut fiber, 2 g L^–1^ salt, 160 mg L^–1^ nitrogen, 190 mg L^–1^ P_2_O_5_, 230 mg L^–1^ K_2_O, pH 6; Werner Tantau GmbH & Co.) and vermiculite (Gebrueder Patzer GmbH & Co.). At 21 days, plants were transferred to a controlled small growth chamber for two additional weeks. Harvests were performed at the end of the light period. The fresh weight (i.e. biomass) and metabolites including amino acids, sugars and TCA-related metabolites, as well as the total protein and starch were measured for every accession in all conditions^42^. These data were used to help obtain accession-specific biomass reactions in a bottom-up assembled model of Arabidopsis metabolism.

We used the Arabidopsis core model covering the major characterized metabolic reactions from primary plant metabolism^9^. The network consists of 407 metabolites and 549 reactions. It includes a biomass reaction denoting the percentage contribution of different metabolites and cellular components to a gram dry weight. Therefore, this synthetic reaction allows us to simulate biomass yield under specific conditions similar to the microbial studies^44^. The network provides three biomass reactions corresponding to optimal N and low N conditions as well as low C condition based on the measurement of soluble metabolites, starch, cell wall precursors, lipid precursors and nucleotides of *A. thaliana* accession Columbia-0 (Col-0) in the three conditions.

We used 67 *A. thaliana* accessions for which there were genotypic data of same coverage available. Altogether, we had access to 214,051 SNPs, here referred to as genome-wide SNPs^19^. To determine the power of consideration of only the genes included in the model, we used only SNPs in the coding regions of the genes included in the model. After filtering the 5% minor allele frequency (MAF) SNPs, GS was conducted with 180,859 genome-wide SNPs and 1824 enzymatic SNPs. To examine the effects of population structure, we also considered the first ten principle components (PCs) of the genome-wide SNPs.

### Reference flux distribution of Col-0

In the following, we avoid consideration of effect of photoperiod which has effects on partitioning of plant resources^45^, and model steady-state growth in the light. A steady-state reference flux distribution, $v^{{\mathrm{Col}}0}$, in Col-0 was obtained by FBA^21^, wherein the flux through the biomass reaction is maximized under the constraints of: (i) steady-state of the model, specified with a stoichiometric matrix ${\mathbf{N}}$, with $m$ rows denoting metabolites and $n$ columns denoting reactions; (ii) lower and upper flux capacities (i.e., bounds); and (iii) bounds on the ratio between the carboxylation and oxygenation reactions catalyzed by RuBisCO to 2.88 and between starch and sucrose synthesis to 2.58^20^. The resulting linear maximization program is as follows:

$${\mathrm{max}}\,v_{{\mathrm{bio}}}^{{\mathrm{Col}}0}$$

s.t.

1 $$\begin{array}{l}{\mathbf{N}} \cdot v^{{\mathrm{Col}}0} = 0,\\ \forall i,1 \le i \le n,\alpha _i \le v_i^{{\mathrm{Col}}0} \le \beta _i,\\ v_{{\mathrm{carboxylation}}}^{{\mathrm{Col}}0} = 2.88v_{{\mathrm{oxygenation}}}^{{\mathrm{Col}}0},\\ v_{{\mathrm{starchsynth}}}^{{\mathrm{Col}}0} = 2.58v_{{\mathrm{sucrosesynth}}}^{{\mathrm{Col}}0},\end{array}$$

where $\alpha _i$ and $\beta _i$ denote the generic lower and upper flux boundaries (−1000 and 1000, respectively, for reversible reactions and 0 and 1000, respectively, for irreversible reactions). This modeling strategy reduces the set of possible flux values while ensuring optimal growth at the biochemical constraints for the selected reactions determining the flux partitioning in carbon primary metabolism. In addition, the imposing of the latter constraints has been shown to lead to prediction about manipulation strategies based on the introduction of photorespiratory bypasses^16^. This optimization program was solved with the help of the COBRA package^46^ in MATLAB.

### Flux distribution of other genotypes

The flux distribution of another genotype Z, $v^{\mathrm{Z}}$, was obtained by minimizing the distance between $v^{\mathrm{Z}}$ and the flux distribution of Col-0, $v^{{\mathrm{Col}}0}$, under the assumption that the genotype minimizes the flux redistribution relative to the fluxes in Col-0. To this end, the Euclidean distance for genotype-specific fluxes of a given reaction was scaled by the reciprocal of the respective flux in Col-0. Therefore, we only considered redistribution of 336 non-zero fluxes in $v^{{\mathrm{Col}}0}$, corresponding to the assumption that genetic variants in an enzyme-coding gene affect the magnitude of non-blocked reactions. The constraint of the ratio between the fluxes of the carboxylation and oxygenation reactions and the ratio between fluxes of starch and sucrose syntheses can vary depending on the photoperiod and genotype^20^. In the absence of information about genotype-specific ratios, we assume the ratios of carboxylation to oxygenation reactions and starch to sucrose syntheses to be bounded in the ranges between 0.94 and 3.81 and between 0.79 and 3.37, respectively, obtained from the measured ratios in Col-0 assuming a variance of (2.88 + 1)/2 and (2.58 + 1)/2.

Two more constraints were added to the optimization program: (i) the genotype-specific biomass reaction and (ii) that the ratio of fluxes through the biomass reaction in Col-0 and genotype Z equals the ratio of measured biomass, $M_{{\mathrm{Col}}0}$ and $M_{\mathrm{Z}}$, respectively. The resulting quadratic program is as follows:

$$\mathop {{{\mathrm{min}}}}\limits_{v^{\mathrm{Z}}} \mathop {\sum }\limits_{i \in {\mathbf{R}}_{ \ne 0}} \left[ {\frac{1}{{v_i^{{\mathrm{Col}}0}}}\left( {v_i^{{\mathrm{Col}}0} - v_i^{\mathrm{Z}}} \right)} \right]^2$$

s.t.

2 $$\begin{array}{l}{\mathbf{N}} \cdot v^{\mathrm{Z}} = 0,\\ \forall i,1 \le i \le n,\alpha _i \le v_i^{\mathrm{Z}} \le \beta _i,\\ 0.94v_{{\mathrm{oxygenation}}}^{\mathrm{Z}} \le v_{{\mathrm{carboxylation}}}^{\mathrm{Z}} \le 3.81v_{{\mathrm{oxygenation}}}^{\mathrm{Z}},\\ 0.79v_{{\mathrm{sucrosesynth}}}^{\mathrm{Z}} \le v_{{\mathrm{starchsynth}}}^{\mathrm{Z}} \le 3.37v_{{\mathrm{sucrosesynth}}}^{\mathrm{Z}},\\ {\mathbf{N}}_{{\mathrm{biomass}}} \leftarrow {\mathbf{N}}_{{\mathrm{biomass}}}^{\mathrm{Z}},\\ v_{{\mathrm{biomass}}}^{\mathrm{Z}} = \frac{{M_{\mathrm{Z}}}}{{M_{{\mathrm{Col}}0}}}v_{{\mathrm{biomass}}}^{{\mathrm{Col}}0} \pm {\it{\epsilon }},\end{array}$$

where ${\mathbf{R}}_{ \ne 0}$ denote the set of reactions with non-zero flux in the reference, ${\mathbf{N}}_{{\mathrm{biomass}}}$ is the column in stoichiometric matrix corresponding to biomass reaction, ${\mathbf{N}}_{{\mathrm{biomass}}}^{\mathrm{Z}}$ is the stoichiometric coefficient from the measurement of genotype Z, and ${\it{\epsilon }}$ is a tunable parameter defined as 90% confidence interval to ensure that the feasible space is non-empty. In addition, the measured biomass values were scaled to fit the range of biomass values that can be obtained with the used model. To this end, for each genotype we first determined the maximum flux through the genotype-specific biomass reaction under the constraints: (i) steady-state, (ii) bounds on the ratio between the carboxylation and oxygenation reactions and between starch and sucrose syntheses, (iii) non-negative carboxylation flux, for biological meaningfulness, and (iv) genotype-specific biomass reaction, by the linear program as Eq. 3:

$$\mathop {{{\mathrm{max}}}}\limits_{v^{\mathrm{Z}}} \,v_{{\mathrm{biomass}}}^{\mathrm{Z}}$$

s.t.

3 $$\begin{array}{l}{\mathbf{N}} \cdot v^{\mathrm{Z}} = 0,\\ \forall i,1 \le i \le n,\alpha _i \le v_i^{\mathrm{Z}} \le \beta _i,\\ 0.94v_{{\mathrm{oxygenation}}}^{\mathrm{Z}} \le v_{{\mathrm{carboxylation}}}^{\mathrm{Z}} \le 3.81v_{{\mathrm{oxygenation}}}^{\mathrm{Z}},\\ 0.79v_{{\mathrm{sucrosesynth}}}^{\mathrm{Z}} \le v_{{\mathrm{starchsynth}}}^{\mathrm{Z}} \le 3.37v_{{\mathrm{sucrosesynth}}}^{\mathrm{Z}},\\ v_{{\mathrm{carboxylation}}}^{\mathrm{Z}} \ge 0,\\ {\mathbf{N}}_{{\mathrm{biomass}}} \leftarrow {\mathbf{N}}_{{\mathrm{biomass}}}^{\mathrm{Z}}.\end{array}$$

We used the average of the maxima over all genotypes to define the model scaling parameter $s_{{\mathrm{model}}} = \overline {v_{{\mathrm{biomass}}}^{{\mathrm{Z}},\,{\mathrm{max}}}}$. To determine the genotype-specific flux distribution by the quadratic program above, we imposed the constraint that $v_{{\mathrm{biomass}}}^{{\mathrm{Z}},\,{\mathrm{ratio}}} = \frac{{M_{\mathrm{Z}}}}{{M_{{\mathrm{Col}}0}}}v_{{\mathrm{biomass}}}^{{\mathrm{Col}}0}$, where $M_{{\mathrm{Col}}0}$ and $M_{\mathrm{Z}}$ are the measured fresh weights of Col-0 and genotype Z, respectively, and $v_{{\mathrm{biomass}}}^{{\mathrm{Col}}0}$ is the biomass flux in the estimated reference flux distribution. Then the measured maximum biomass was defined as the maximum value among all genotypes, $s_{{\mathrm{measurement}}} = {\mathrm{max}}(v_{{\mathrm{biomass}}}^{{\mathrm{all}},\,{\mathrm{ratio}}})$, and is referred as the measurement scaling parameter. To ensure the feasibility of our approach, the biomass flux in the genotype Z in the optimization program (Eq. 2) was finally scaled by the two scaling parameters above, as $v_{{\mathrm{biomass}}}^{{\mathrm{Z}},\,{\mathrm{scale}}} = \frac{{(s_{{\mathrm{model}}} - \delta )}}{{s_{{\mathrm{measurement}}}}}v_{{\mathrm{biomass}}}^{{\mathrm{Z}},\,{\mathrm{ratio}}}$, where $\delta$ is a tunable parameter, here set of the value of 1.1 × 10^−4^. All quadratic programs were solved with the help of the cvx package in MATLAB^47^.

The genotype-specific biomass reaction was determined as in the Arabidopsis core model reconstruction^9^. In total 30 soluble metabolites including free amino acids, sugars, TCA-related metabolites and others, as well as starch, were measured in all accessions and converted into the unit of μmol per gram dry weight. The protein-bound amino acids were calculated from the fraction of 20 amino acids in the total protein concentration for every accession. Because there are no genotype-specific measurements available for cell wall precursors, lipid precursors, nucleotides and ATP, we assumed them to be the same for all accessions (the same values in every row of Supplementary Data 1). Differences between growth of the accessions, used as constraints, compensate for keeping the coefficients of these components of biomass the same across the accessions.

### From statistical models of fluxes to predicted biomass

We note that the predicted genotype-specific flux distributions provide the flux value across all accessions for each reaction in the analyzed model. The flux of each reaction ${\mathrm{R}}_i$ is modeled according to classical GS based on the given set of SNPs. This resulted in the function $g_i\left( \cdot \right)$ for the training set, which was in turn used to obtain a predicted flux GEBV for the testing set. Statistical modeling is successful in the case where the trait shows variability around a single mode. To this end, all non-zero fluxes were modeled using the state-of-art method for GS, ridge regression Best Linear Unbiased Prediction (rrBLUP)^4^. rrBLUP is based on a linear mixed model that can be simultaneously estimated from genome-wide SNPs.

Since the flux distribution resulting from the functions $g_i\left( \cdot \right)$, evaluated from a given set of SNPs, $S_Z$, of a genotype Z, may not be at steady-state, we determine the closest steady-state flux distribution, $w^{\mathrm{Z}}$, in a similar minimization program, given below:

$$\mathop {{{\mathrm{min}}}}\limits_{w^{\mathrm{Z}}} \mathop {\sum }\limits_{i \in {\mathbf{R}}_{ \ne 0}} \left[ {\frac{1}{{g_i\left( {S_{\mathrm{Z}}} \right)}}\left( {w_i^{\mathrm{Z}} - g_i\left( {S_{\mathrm{Z}}} \right)} \right)} \right]^2$$

s.t.

4 $$\begin{array}{l}{\mathbf{N}} \cdot w^{\mathrm{Z}} = 0,\\ \forall i,1 \le i \le n,\alpha _i \le w_i^{\mathrm{Z}} \le \beta _i,\\ 0.94w_{{\mathrm{oxygenation}}}^{\mathrm{Z}} \le w_{{\mathrm{carboxylation}}}^{\mathrm{Z}} \le 3.81w_{{\mathrm{oxygenation}}}^{\mathrm{Z}},\\ 0.79w_{{\mathrm{sucrosesynth}}}^{\mathrm{Z}} \le w_{{\mathrm{starchsynth}}}^{\mathrm{Z}} \le 3.37w_{{\mathrm{sucrosesynth}}}^{\mathrm{Z}},\\ {\mathbf{N}}_{{\mathrm{biomass}}} \leftarrow {\mathbf{N}}_{{\mathrm{biomass}}}^{\mathrm{Z}},\\ w_{{\mathrm{biomass}}}^{\mathrm{Z}} \ge 0.\end{array}$$

Instead of constraining the biomass flux to the ratio of measured biomass, this program only constrained the biomass flux to be positive. Thus, the resulting flux distribution contains the entry for $w_{{\mathrm{biomass}}}^{\mathrm{Z}}$, which we use as GEBV for biomass resulting from our approach. To determine the predictability of the approach, we conducted 3-fold cross-validation with 50 repetitions to obtain the mean correlation coefficient between measured and predicted flux through the biomass reaction. GS modeling and predictions were conducted in the R programming environment using the rrBLUP package^26^. For comprehensive comparative analysis, was also used the BayesC models, obtained by using the R package BGLR (Bayesian Generalized Linear Regression)^48^.

### Transferability of the approach to an unseen environment

The developed flux models in environment E_1_ may have poor performance in another environment E_2_ due to the usually large genotype-by-environment interaction observed for yield-related traits^49^. We extended our approach to allow for prediction of flux and biomass GEBV across environments. To this end, we propose an approach which relies on the reference flux distribution of Col-0 in two environments. Again, given the flux distribution, $v^{{\mathrm{Col}}0,{\mathrm{E}}_1}$, of Col-0 in environment E_1_, we obtain the flux distribution, $v^{{\mathrm{Col}}0,{\mathrm{E}}_2}$, in environment E_2_ under the assumption that it minimizes the distance while ensuring that (i) the Col-0 biomass reaction in E_2_ and (ii) the ratio of measured biomasses coincides with the ratio of biomass fluxes in two environments. This can be obtained by solving the following quadratic program:

$$\mathop {{{\mathrm{min}}}}\limits_{v^{{\mathrm{Col}}0,{\mathrm{E}}_2}} \mathop {\sum }\limits_{i \in {\mathbf{R}}_{ \ne 0}} \left[ {\frac{1}{{v_i^{{\mathrm{Col}}0,\,{\mathrm{E}}_1}}}\left( {v_i^{{\mathrm{Col}}0,\,{\mathrm{E}}_1} - v_i^{{\mathrm{Col}}0,\,{\mathrm{E}}_2}} \right)} \right]^2$$

s.t.

5 $$\begin{array}{l}{\mathbf{N}} \cdot v^{{\mathrm{Col}}0,{\mathrm{E}}_2} = 0,\\ \forall i,1 \le i \le n,\alpha _i \le v_i^{{\mathrm{Col}}0,{\mathrm{E}}_2} \le \beta _i,\\ 0.94v_{{\mathrm{oxygenation}}}^{{\mathrm{Col}}0,{\mathrm{E}}_2} \le v_{{\mathrm{carboxylation}}}^{{\mathrm{Col}}0,{\mathrm{E}}_2} \le 3.81v_{{\mathrm{oxygenation}}}^{{\mathrm{Col}}0,{\mathrm{E}}_2},\\ 0.79v_{{\mathrm{sucrosesynth}}}^{{\mathrm{Col}}0,{\mathrm{E}}_2} \le v_{{\mathrm{starchsynth}}}^{{\mathrm{Col}}0,{\mathrm{E}}_2} \le 3.37v_{{\mathrm{sucrosesynth}}}^{{\mathrm{Col}}0,{\mathrm{E}}_2},\\ {\mathbf{N}}_{{\mathrm{biomass}}} \leftarrow {\mathbf{N}}_{{\mathrm{biomass}}}^{{\mathrm{Col}}0,{\mathrm{E}}_2},\\ v_{{\mathrm{biomass}}}^{{\mathrm{Col}}0,{\mathrm{E}}_2} = \frac{{M_{{\mathrm{Col}}0,{\mathrm{E}}_2}}}{{M_{{\mathrm{Col}}0,{\mathrm{E}}_1}}}v_{{\mathrm{biomass}}}^{{\mathrm{Col}}0,{\mathrm{E}}_1} \pm {\it{\epsilon }},\end{array}$$

where ${\mathbf{N}}_{{\mathrm{biomass}}}^{{\mathrm{Col}}0,{\mathrm{E}}_2}$ is the stoichiometric coefficient from the measurement of Col-0 in environment E_2_ and $M_{{\mathrm{Col}}0,{\mathrm{E}}_1}$ and $M_{{\mathrm{Col}}0,{\mathrm{E}}_2}$ are the measured biomass of Col-0 in environment E_1_ and E_2_, respectively.

Let ${\mathbf{P}}$ be a subset of exchange reactions whereby the organism exchanges molecules with the environment. Given $v^{{\mathrm{Col}}0,{\mathrm{E}}_1}$ and $v^{{\mathrm{Col}}0,{\mathrm{E}}_2}$, we can obtain the flux ratio for each reaction in ${\mathbf{P}}$ between the two environments. To obtain the flux distribution $w^{{\mathrm{Z}},{\mathrm{E}}_2}$ for genotype Z in the unseen environment E_2_, given the flux GEBVs $g\left( {S_{\mathrm{Z}}} \right)$ and the flux ratios for the exchange reactions in ${\mathbf{P}}$ from $v^{{\mathrm{Col}}0,{\mathrm{E}}_1}$ and $v^{{\mathrm{Col}}0,{\mathrm{E}}_2}$, we solve the following quadratic program:

$$\mathop {{{\mathrm{min}}}}\limits_{w^{{\mathrm{Z}},{\mathrm{E}}_2}} \mathop {\sum }\limits_{i \in {\mathbf{R}}_{ \ne 0}} \left[ {\frac{1}{{g_i\left( {S_{\mathrm{Z}}} \right)}}\left( {w_i^{{\mathrm{Z}},{\mathrm{E}}_2} - g_i\left( {S_{\mathrm{Z}}} \right)} \right)} \right]^2$$

s.t.

6 $$\begin{array}{l}{\mathbf{N}} \cdot w^{{\mathrm{Z}},{\mathrm{E}}_2} = 0,\\ \forall i,1 \le i \le n,\alpha _i \le w_i^{{\mathrm{Z}},{\mathrm{E}}_2} \le \beta _i,\\ 0.94w_{{\mathrm{oxygenation}}}^{{\mathrm{Z}},{\mathrm{E}}_2} \le w_{{\mathrm{carboxylation}}}^{{\mathrm{Z}},{\mathrm{E}}_2} \le 3.81w_{{\mathrm{oxygenation}}}^{{\mathrm{Z}},{\mathrm{E}}_2},\\ 0.79w_{{\mathrm{sucrosesynth}}}^{{\mathrm{Z}},{\mathrm{E}}_2} \le w_{{\mathrm{starchsynth}}}^{{\mathrm{Z}},{\mathrm{E}}_2} \le 3.37w_{{\mathrm{sucrosesynth}}}^{{\mathrm{Z}},{\mathrm{E}}_2},\\ {\mathbf{N}}_{{\mathrm{biomass}}} \leftarrow {\mathbf{N}}_{{\mathrm{biomass}}}^{{\mathrm{Z}},{\mathrm{E}}_1},\\ w_{{\mathrm{biomass}}}^{{\mathrm{Z}},{\mathrm{E}}_2} \ge 0,\\ \forall j \in {\mathbf{P}},v_j^{{\mathrm{Z}},{\mathrm{E}}_2} = \frac{{v_j^{{\mathrm{Col}}0,{\mathrm{E}}_2}}}{{v_j^{{\mathrm{Col}}0,{\mathrm{E}}_1}}}g_j\left( {S_{\mathrm{Z}}} \right) \pm {\it{\epsilon }}.\end{array}$$

We note that the genotype-specific biomass reaction used in this program is from the measurement in environment E_1_. For prediction in an unseen environment, we additionally require only access to a reference genotype-specific biomass reaction from environment E_2_. In the program, we considered the exchange reactions of photon, CO_2_, H_2_O and NO_3_ to belong to the set ${\mathbf{P}}$ when predicting from optimal N to low N condition, and the exchange reaction of CO_2_ when predicting from optimal N to low C condition. Similarly, the predictability was determined by the mean correlation coefficient between measured and predicted biomass using 3-fold cross-validation with 50 repetitions.

The differences in the effect of the environment on a genotype may in part be due to the presence of genotype-environment interactions. Our approach accounts for such differences since the last program does not impose that all genotypes respond in the same fashion to the environmental change, particularly not with respect to their internal fluxes. The reason is that the statistical model for the fluxes, $w^{{\mathrm{Z}},{\mathrm{E}}_2}$, based on the genotypic data, can take a particular direction for a specific genotype when imposing the steady-state and other constraints.

### Robustness of Col-0 flux distributions

To test the robustness of the reference genome flux distribution, we randomly sample 50 values $v_i^{\mathrm{r}}$ for each reaction $i$ from the respective variance interval $\left[ {v_i^{{\mathrm{Col}}0} - v_i^{{\mathrm{Col}}0} \times e,v_i^{{\mathrm{Col}}0} + v_i^{{\mathrm{Col}}0} \times e} \right]$ resulting in the set of sampled reference flux distributions $v^{\mathrm{r}},r = 1, \ldots ,50$, $e$ is the variance percentage. The sampled flux distributions, however, might not comply with the physio-chemical and steady-state constraints. Therefore, we use a minimization program to obtain the steady-state flux distribution $v^{{\mathrm{Col}}0,{\mathrm{r}}}$ closest to a sampled flux distribution $v^{\mathrm{r}}$. The ratio between fluxes of the carboxylation and oxygenation reactions and between starch and sucrose syntheses, as well as the biomass flux, are constrained to the values in the original reference flux distribution. The quadratic program is given in the following Eq. 7:

$$\mathop {{{\mathrm{min}}}}\limits_{v^{{\mathrm{Col}}0,{\mathrm{r}}}} \mathop {\sum }\limits_{i \in {\mathbf{R}}_{ \ne 0}} \left[ {\frac{1}{{v_i^{\mathrm{r}}}}\left( {v_i^{\mathrm{r}} - v_i^{{\mathrm{Col}}0,{\mathrm{r}}}} \right)} \right]^2$$

s.t.

7 $$\begin{array}{l}{\mathbf{N}} \cdot v^{{\mathrm{Col}}0,{\mathrm{r}}} = 0,\\ \forall i,1 \le i \le n,\alpha _i \le v_i^{{\mathrm{Col}}0,{\mathrm{r}}} \le \beta _i,\\ v_{{\mathrm{carboxylation}}}^{{\mathrm{Col}}0,{\mathrm{r}}} = 2.88v_{{\mathrm{oxygenation}}}^{{\mathrm{Col}}0,{\mathrm{r}}} + {\it{\epsilon }},\\ v_{{\mathrm{starchsynth}}}^{{\mathrm{Col}}0,{\mathrm{r}}} = 2.58v_{{\mathrm{sucrosesynth}}}^{{\mathrm{Col}}0,{\mathrm{r}}} + {\it{\epsilon }},\\ v_{{\mathrm{biomass}}}^{{\mathrm{Col}}0,{\mathrm{r}}} = v_{{\mathrm{biomass}}}^{{\mathrm{Col}}0},\end{array}$$

where ${\it{\epsilon }}$ is a tunable parameter, here set of the value of 10^−4^. For the reason that we observed very small variance between 50 random flux distributions, the means of the resulting flux distributions were used as a reference flux distribution in the netGS approach to determine the robustness of the predictions.

### Reporting summary

Further information on research design is available in the Nature Research Reporting Summary linked to this article.

## Supplementary information

Supplementary Information

Peer Review File

Reporting Summary

Description of Additional Supplementary Files

Supplementary Data 1

Supplementary Data 2

Supplementary Data 3

Supplementary Data 4

Supplementary Data 5

Supplementary Data 6

Supplementary Data 7

Supplementary Data 8

Supplementary Data 9

Supplementary Data 10

Supplementary Data 11

Supplementary Data 12

Supplementary Data 13

Supplementary Data 14

Supplementary Data 15

Supplementary Data 16

Supplementary Software 1

## Data Availability

Data supporting the findings of this work are available within the paper and its Supplementary Information files. A reporting summary for this Article is available as a Supplementary Information file. The datasets generated and analyzed during the current study are available from the corresponding author upon request. The source data underlying Figs. 2 and 3, and Supplementary Figs. 2–7 are provided as a Source Data file.

## Source data

Source Data

## Code availability

The R and Matlab code of netGS approach can be found in Supplementary Software 1 or at https://github.com/Hao-Tong/netGS.
