# Supplementary material for: Integrating molecular markers into metabolic models improves genomic selection for Arabidopsis growth
Source: Nat Commun. 2020 May 15;11:2410. doi: 10.1038/s41467-020-16279-5 (PMC7229213; doi:10.1038/s41467-020-16279-5)
Supplement: Supplementary file 1 — Supplementary Information [file 41467_2020_16279_MOESM1_ESM.pdf]

Integrating molecular markers into metabolic models improves genomic selection for Arabidopsis growth

Tong *et al.*

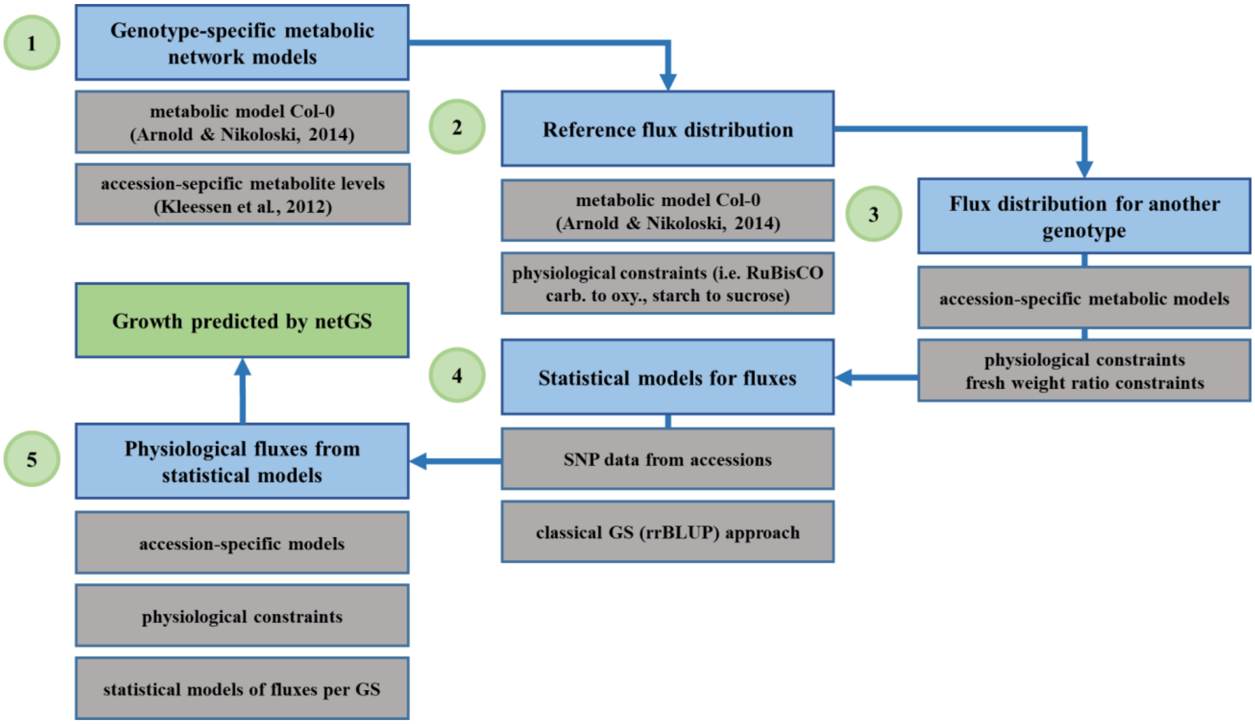

Supplementary Figure 1. Graphical representation of netGS steps. NetGS includes five key steps: (1) genotype-specific metabolic network generation, (2) prediction of a steady-state flux distribution for a reference genotype, (3) prediction of steady-state flux distribution for all genotypes in a given genetic population, (4) obtaining statistical models for fluxes based on SNPs, and (5) prediction of physiological flux distribution from statistical models; this is achieved by finding the closest steady-state flux distribution to that obtained from statistical models. The flux of the biomass reaction in the final step is used to predict growth.

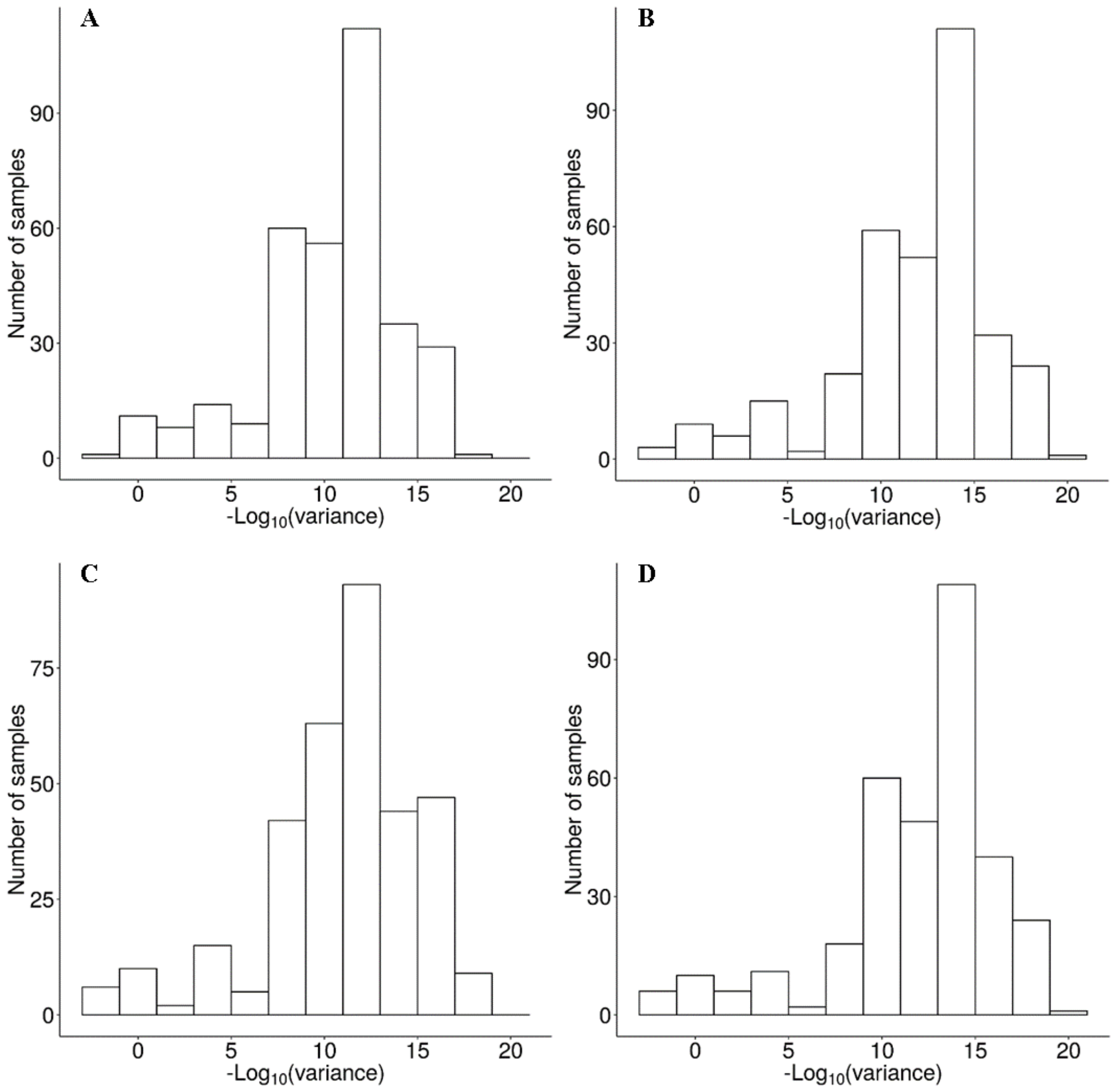

Supplementary Figure 2. The distribution of variance of fluxes in robustness test. In total, 50 random flux values are sampled for n=336 reactions from the respective variance interval with 5% (**A**), 10% (**B**), 15% (**C**), and 20% (**D**) of the reference flux distribution and the closest steady-state flux distributions are determined. The negative log-transformed variances for each flux over the 50 resulting steady-state flux distributions are shown in the respective histograms. Source data are provided as a Source Data file.

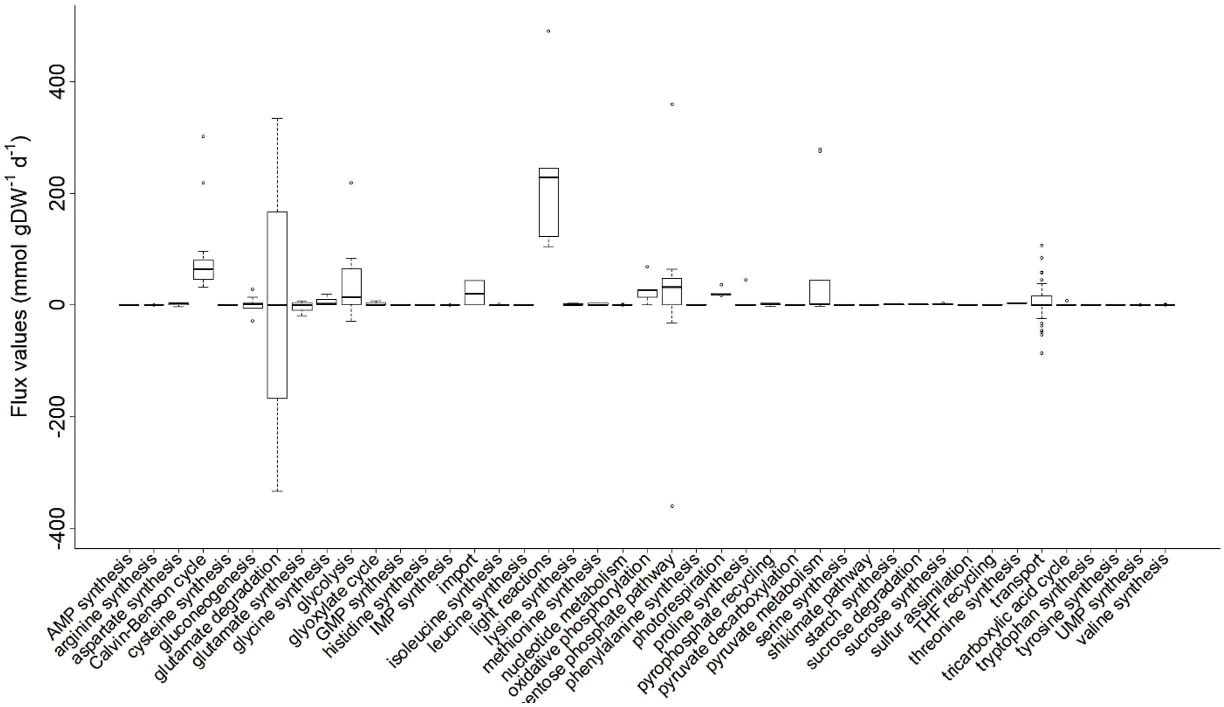

Supplementary Figure 3. Flux ranges of all metabolic systems in the metabolic model. The metabolic systems in the model of central metabolism are obtained from the original model for Col-0. Only 12 of these, namely: the Calvin-Benson cycle, photosynthesis light reactions, glycolysis, oxidative phosphorylation, pentose phosphate pathway, gluconeogenesis, glutamate synthesis and degradation, glycine synthesis, pyruvate metabolism, and import and transport reactions exhibit large variation, and the remaining systems contain reactions of negligible fluxes. n=3 to 76 fluxes in each metabolic system are used. Center line, median; box limits, 75th and 25th quartiles; whiskers, 1.5×interquartile range; points, outliers. Source data are provided as a Source Data file.

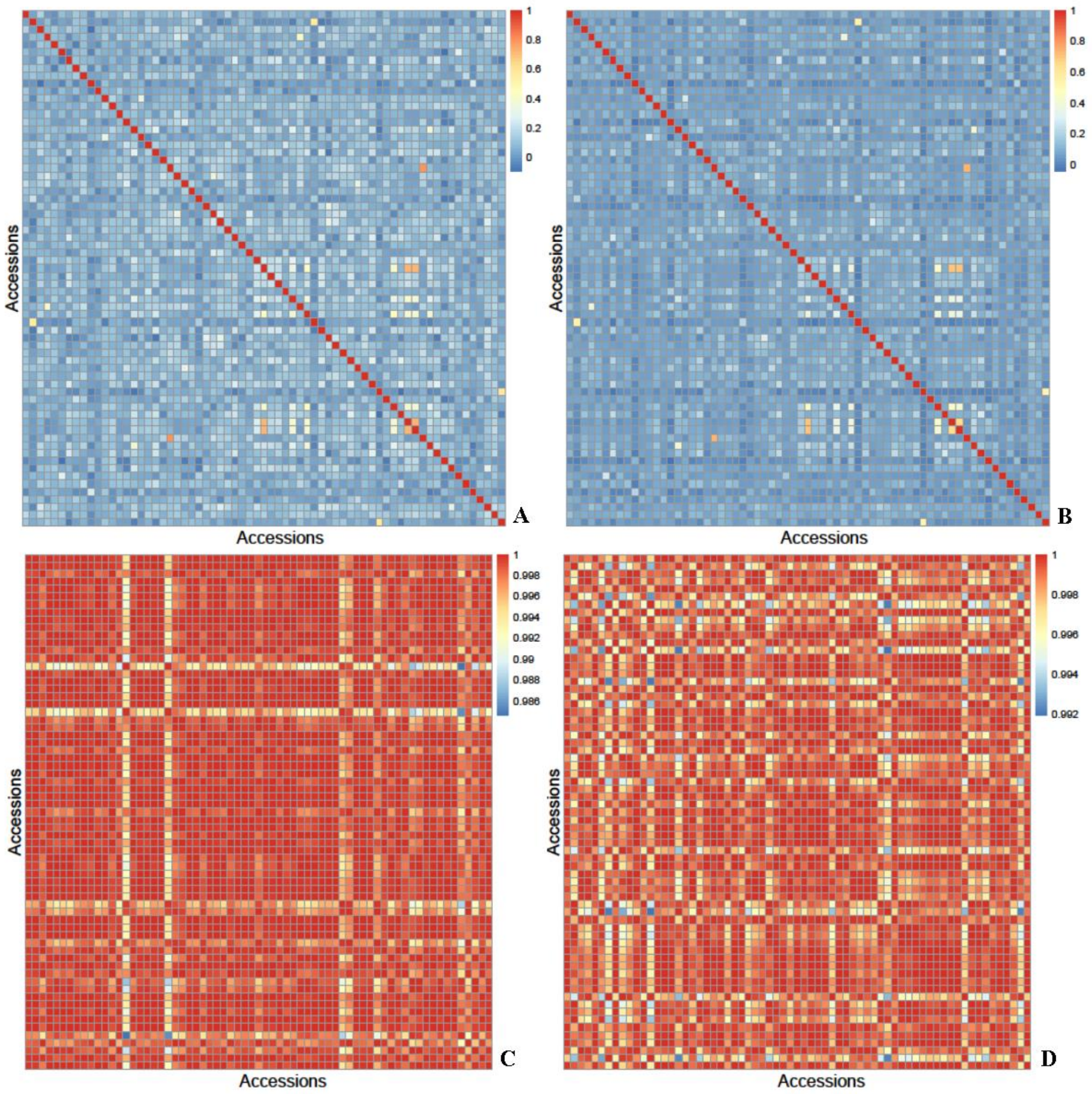

Supplementary Figure 4. Heatmaps of the correlation between accessions on different data. Four heatmaps represent Pearson correlation coefficient using enzymatic SNPs (**A**), Pearson correlation coefficient using genome-wide SNPs (**B**), Pearson correlation coefficient using metabolite levels (**C**), and Pearson correlation coefficient using predicted flux distributions (**D**), respectively. Source data are provided as a Source Data file.

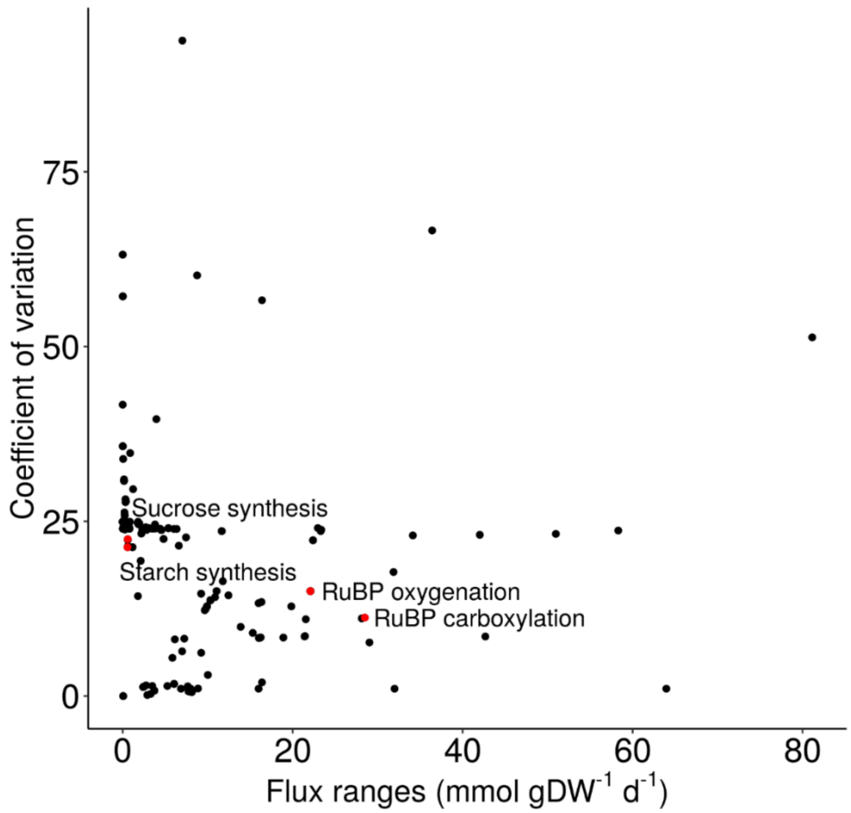

Supplementary Figure 5. Variability of fluxes over the examined *A. thaliana* accessions. Variability of fluxes is quantified by the flux range (i.e. the difference of the maximum and minimum flux value) and the coefficient of variation (i.e. the ratio of the standard deviation to the mean) over the investigated accessions. All values shown are absolute values. The majority of reactions exhibit coefficients of variation greater than 10%, indicating suitability for statistical modeling. Red dots correspond to sucrose synthesis, starch synthesis, RuBisCO oxygenation and RuBisCO carboxylation reactions employed as constraints in the mathematical programs to determine the genotype-specific flux distributions. Source data are provided as a Source Data file.

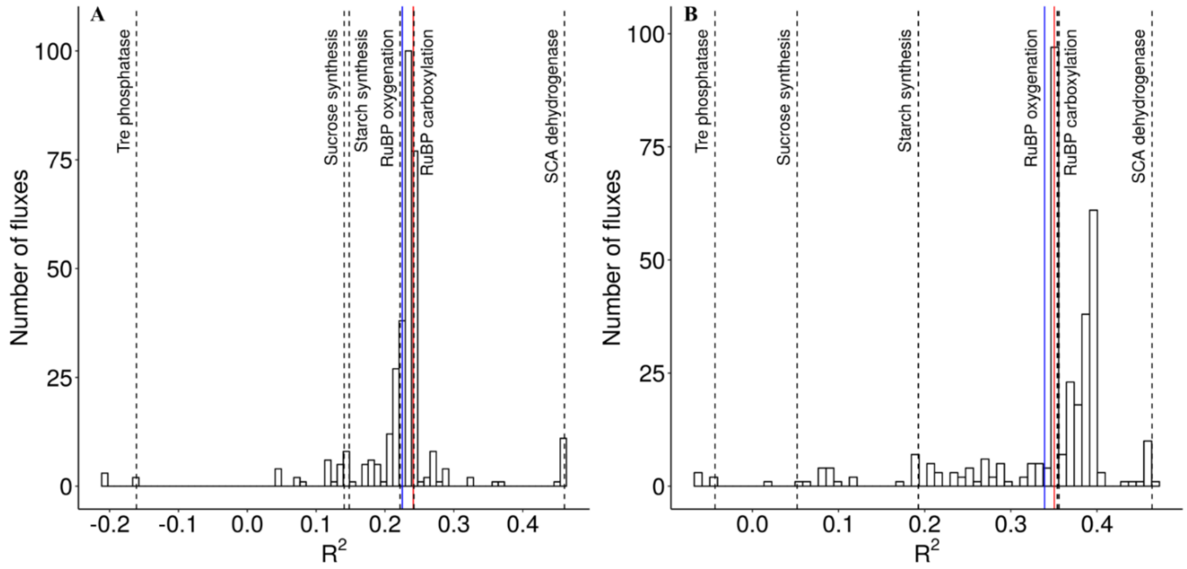

Supplementary Figure 6. The distribution of prediction accuracies for fluxes using rrBLUP. The predictions for flux are performed with enzymatic SNPs (**A**) and genome-wide SNPs (**B**) with 150 cross-validations (i.e. 50 repetitions of 3-fold cross-validation). X-axis shows the averaged correlation coefficient between flux and predicted flux from rrBLUP model, y-axis shows the corresponding number of fluxes. Red vertical line indicates the prediction accuracy for growth (i.e. flux of the biomass reaction), blue vertical line indicates the averaged prediction accuracy for flux over all fluxes, the dashed vertical line indicates the prediction accuracy for the showed flux. Source data are provided as a Source Data file.

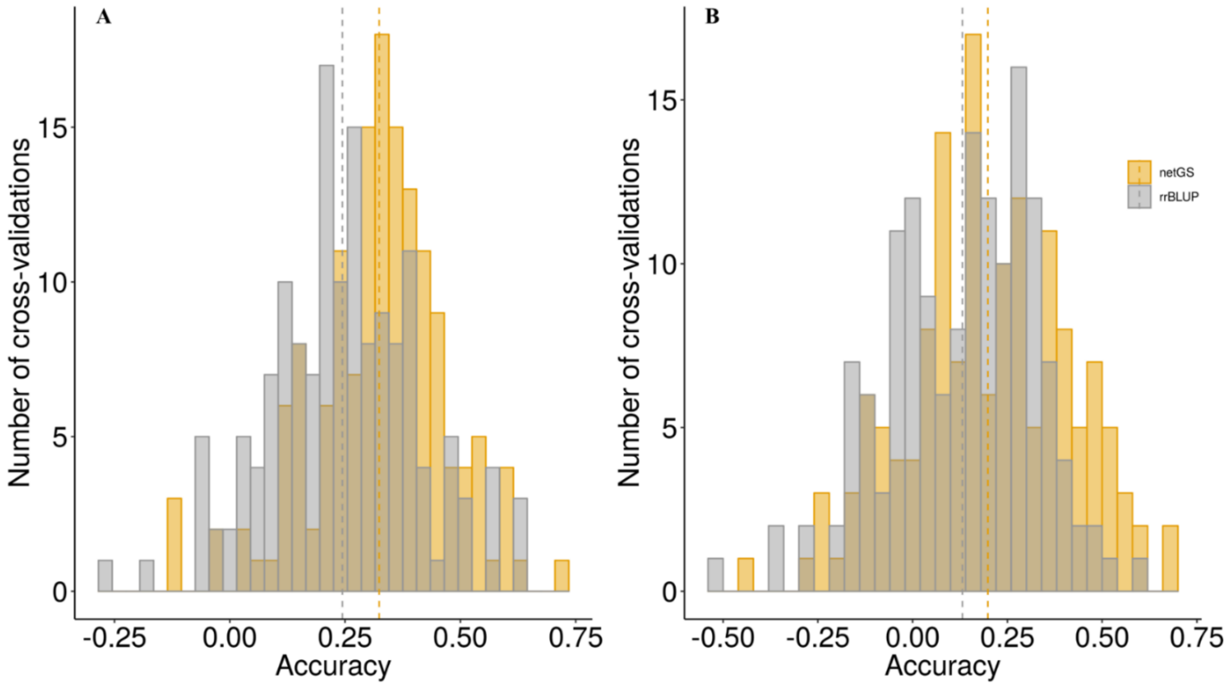

Supplementary Figure 7. The distribution of prediction accuracies for growth using netGS. The predictions for growth are performed using enzymatic SNPs with 150 cross-validations (i.e. 50 repetitions of 3-fold cross-validation) within optimal N condition (**A**) and from optimal N to low N condition (**B**). X-axis shows the correlation coefficient between measured and predicted biomass, y-axis shows the corresponding number of cross-validations. The grey bar indicates prediction accuracy using rrBLUP and the orange bar indicates prediction accuracy using netGS. Source data are provided as a Source Data file.

Supplementary Table 1. Summary of the key plant-specific reactions.

Reaction names	Reference	Min	Max	Mean
RuBisCO carboxylation	73.150	45.215	73.698	59.194
RuBisCO oxygenation	25.432	24.258	46.348	36.780
Starch synthesis	0.921	0.320	0.892	0.595
Sucrose synthesis	0.204	0.239	0.836	0.587
Carboxylation/Oxygenation	2.880	0.985	3.026	1.678
Starch/Sucrose	2.580	0.971	2.984	1.044

Note: The table shows the summary of RuBisCO carboxylation and oxygenation fluxes and their ratio as well as starch and sucrose syntheses and their ratio. The fluxes in reference accession (Col-0) together with the minimum, the maximum, and the mean values over all examined accessions are presented. The units of all fluxes are mmol gDW^{-1} d^{-1}.

Supplementary Table 2. The Mantel correlation between correlation matrices.

**Matrices**	**Enzymatic SNPs**	**Genome-wide SNPs**	**Metabolites**	**Fluxes**
Enzymatic SNPs	.	0.942	0.065	0.116
Genome-wide SNPs	8.99E-033	.	0.082	0.118
Metabolites	0.600	0.511	.	-0.073
Fluxes	0.349	0.342	0.559	.

Note: Four correlation matrices were determined based on enzymatic SNPs, genome-wide SNPs, metabolite levels, and flux distributions of accessions. The upper triangular matrix contains the Mantel correlation coefficients and the lower triangular matrix includes the corresponding p-values for the four correlation matrices.

Supplementary Table 3. Threshold-dependent prediction accuracies for growth using netGS.

Scenarios	Thresholds	Flux number	Accuracy	Increase (%)
1	-	336	0.313	28.2
2	0	298.68	0.312	27.9
3	0.1	263.19	0.317	29.7
4	0.2	193.20	0.315	29.0
5	0.3	113.63	0.302	23.5
6	biomass	172.43	0.324	32.6

Note: The number of fluxes, mean accuracy, and relative increase to rrBLUP are shown for each threshold value. The thresholds used include flux estimations with accuracy greater than 0, 0.1, 0.2 and 0.3, as well as those with accuracy values higher than the prediction accuracy for growth in rrBLUP (i.e. flux of the biomass reaction). The netGS are performed using enzymatic SNPs within optimal N condition with 150 cross-validations (i.e. 50 repetitions of 3-fold cross-validation)

Supplementary Table 4. The summary of exchange reactions in the three conditions.

Flux names	Min	Max	Mean	Optimal N	Low N		Low C	
					Value	Ratio	Value	Ratio
Import Photon	935.88	999.85	979.95	1000	979.53	0.98	975.19	0.98
Import CO_2	23.75	65.77	43.57	64.44	40.91	0.63	33.20	0.52
Import H_2O	19.37	53.51	35.53	52.52	33.86	0.64	26.93	0.51
Import Pi	0.014	0.043	0.027	0.040	0.021	0.52	0.022	0.55
Import NO_3	3.08	8.46	5.64	8.11	6.28	0.77	4.07	0.50
Import SO_4	0.077	0.206	0.140	0.202	0.152	0.75	0.101	0.50
Export O_2	29.44	80.40	53.65	78.82	52.87	0.67	40.28	0.51

Note: The minimum, maximum, and mean value of exchange reactions over all examined accessions estimated under optimal N condition are presented. The exchange reactions of the reference accession (Col-0) in optimal N, low N, and low C conditions, together with the ratios with respect to optimal N condition are presented as well. The units of all fluxes are mmol gDW^{-1} d^{-1}.
