# Supplementary material for: Integrating molecular markers into metabolic models improves genomic selection for Arabidopsis growth
Source: Nat Commun. 2020 May 15;11:2410. doi: 10.1038/s41467-020-16279-5 (PMC7229213; doi:10.1038/s41467-020-16279-5)
Supplement: Supplementary file 4 — Description of Additional Supplementary Files [file 41467_2020_16279_MOESM4_ESM.docx]

**Description of Additional Supplementary Files**

File name: Supplementary Data 1

Description: Condition and accession-specific biomass reactions. The coefficients in biomass reactions of Col-0 in three conditions and all other accessions in optimal N condition in the studied metabolic model are shown in the table. The calculation of coefficients followed the same procedure used in generating the metabolic network of Col-0 in the AraCore metabolic model.

File name: Supplementary Data 2

Description: Accession-specific steady-state flux distributions. The fluxes of 336 reactions (with non-zero fluxes in Col-0) used in the netGS modeling are presented in the reference accession (Col-0) and the 67 accessions in genetic population. The mean values for each flux over all examined accessions are also presented. The table also includes a list of reactions of zero-flux in a separate sheet.

File name: Supplementary Data 3

Description: Robustness test of flux distribution in reference accession (Col-0). In total, 50 random flux values are sampled for 336 reactions from the respective variance interval with 5%, 10%, 15% and 20% of the reference flux distribution and the closest steady-state flux distributions are determined. The mean value and variance of all flux distributions in each scenario are presented in the table.

File name: Supplementary Data 4

Description: Validation of estimated fluxes based on maximal rates. The maximal rates and predicted fluxes in all examined accessions are shown in the table. We find slightly larger predicted fluxes than maximal rates only for nitrate reductase in 30 out of the 67 accessions. The fluxes of reactions catalyzed by the remaining five enzymes are consistently smaller than the respective accession-specific maximal rates.

File name: Supplementary Data 5

Description: Summary of accession-specific flux distributions. The minimum, maximum, mean, and median, as well as the standard deviation and coefficient of variation of each flux over all examined accessions are presented.

File name: Supplementary Data 6

Description: The prediction accuracies for fluxes using rrBLUP. The averaged correlation coefficient of each flux and predicted flux using enzymatic and genome-wide SNPs with 150 cross-validations (i.e. 50 repetitions of 3-fold cross-validation) are provided in the table. The correlation coefficients (R^2^) between each flux and measured biomass are shown as well.

File name: Supplementary Data 7

Description: The prediction accuracies for growth within optimal N condition using rrBLUP and netGS. Three approaches: rrBLUP of fresh weight, netGS without accession-specific biomass reaction, and netGS with accession-specific biomass reaction are applied, for comparison, using enzymatic and genome-wide SNPs. The prediction accuracies of 150 cross-validations (i.e. 50 repetitions of 3-fold cross-validation) and the mean values of these are shown in the table. In addition, the coancestry coefficients for each cross-validation are presented as the measurement of genetic distance between the training and testing populations.

File name: Supplementary Data 8

Description: The prediction accuracies for growth in robustness test. The robustness test is performed by random sampling within a specified relative error (5%, 10%, 15% and 20%). The netGS approach with accession-specific biomass reaction is then applied within optimal N condition and from optimal N to low N condition using enzymatic and genome-wide SNPs. The prediction accuracies of 150 cross-validations (i.e. 50 repetitions of 3-fold cross-validation) and the mean values of these are shown in the table.

File name: Supplementary Data 9

Description: The prediction accuracies for growth from optimal N to low N condition using rrBLUP and netGS. Three approaches: rrBLUP of fresh weight, netGS without accession-specific biomass reaction, and netGS with accession-specific biomass reaction are applied, for comparison, using enzymatic and genome-wide SNPs. The prediction accuracies of 150 cross-validations (i.e. 50 repetitions of 3-fold cross-validation) and the mean values of these are shown in the table. The NA denotes the predicted biomass is constant over all accessions in testing population.

File name: Supplementary Data 10

Description: The prediction accuracies for growth using BayesC and netGS via BayesC within optimal N condition. Four approaches: BayesC of fresh weight, netGS via BayesC without accession-specific biomass reaction, netGS via BayesC with accession-specific biomass reaction, and robustness test by random sampling within a specified relative error are applied, for comparison, using enzymatic SNPs. The prediction accuracies of 150 cross-validations (i.e. 50 repetitions of 3-fold cross-validation) and the mean values of these are shown in the table.

File name: Supplementary Data 11

Description: The prediction accuracies for growth using BayesC and netGS via BayesC from optimal N to low N condition. Four approaches: BayesC of fresh weight, netGS via BayesC without accession-specific biomass reaction, netGS via BayesC with accession-specific biomass reaction, and robustness test by random sampling within a specified relative error are applied, for comparison, using enzymatic SNPs. The prediction accuracies of 150 cross-validations (i.e. 50 repetitions of 3-fold cross-validation) and the mean values of these are shown in the table.

File name: Supplementary Data 12

Description: The prediction accuracies for growth including population structure within optimal N condition. Four approaches: rrBLUP or BayesC of fresh weight, netGS without accession-specific biomass reaction, netGS with accession-specific biomass reaction, and robustness test by random sampling within a specified relative error are applied for comparison. rrBLUP-related models are performed with enzymatic and genome-wide SNPs, BayesC-related models are performed with only enzymatic SNPs due to the time consumption. The prediction accuracies of 150 cross-validations (i.e. 50 repetitions of 3-fold cross-validation) and the mean values of these are shown in the table.

File name: Supplementary Data 13

Description: The prediction accuracies for growth including population structure from optimal N to low N condition. Four approaches: rrBLUP or BayesC of fresh weight, netGS without accession-specific biomass reaction, netGS with accession-specific biomass reaction, and robustness test by random sampling within a specified relative error are applied for comparison. rrBLUP-related models are performed with enzymatic and genome-wide SNPs, BayesC-related models are performed with only enzymatic SNPs due to the time consumption. The prediction accuracies of 150 cross-validations (i.e. 50 repetitions of 3-fold cross-validation) and the mean values of these are shown in the table.

File name: Supplementary Data 14

Description: The prediction accuracies for growth using pFBA within optimal N condition. Four approaches: rrBLUP of fresh weight, netGS using pFBA without accession-specific biomass reaction, netGS using pFBA with accession-specific biomass reaction, and robustness test using pFBA by random sampling within a specified relative error are applied, for comparison, using enzymatic and genome-wide SNPs. The prediction accuracies of 150 cross-validations (i.e. 50 repetitions of 3-fold cross-validation) and the mean values of these are shown in the table.

File name: Supplementary Data 15

Description: The prediction accuracies for growth using pFBA from optimal N to low N condition. Four approaches: rrBLUP of fresh weight, netGS using pFBA without accession-specific biomass reaction, netGS using pFBA with accession-specific biomass reaction, and robustness test using pFBA by random sampling within a specified relative error are applied, for comparison, using enzymatic and genome-wide SNPs. The prediction accuracies of 150 cross-validations (i.e. 50 repetitions of 3-fold cross-validation) and the mean values of these are shown in the table. The NA denotes the results are infeasible in the approach of netGS without accession-specific biomass reaction.

File name: Supplementary Data 16
Description: Description: The prediction accuracies for growth from optimal N to low C condition using rrBLUP and netGS. Four approaches: rrBLUP of fresh weight, netGS without accession-specific biomass reaction, netGS with accession-specific biomass reaction, and robustness test by random sampling within a specified relative error are applied, for comparison, using enzymatic and genome-wide SNPs. The prediction accuracies of 150 cross-validations (i.e. 50 repetitions of 3-fold cross-validation) and the mean values of these are shown in the table.

File name: Supplementary Software 1

Description: The R and Matlab code with examples of netGS approach.
