# Supplementary material for: Integrating molecular markers into metabolic models improves genomic selection for Arabidopsis growth
Source: Nat Commun. 2020 May 15;11:2410. doi: 10.1038/s41467-020-16279-5 (PMC7229213; doi:10.1038/s41467-020-16279-5)
Supplement: Supplementary file 21 — Supplementary Software 1 [file 41467_2020_16279_MOESM21_ESM.zip › Readme.docx]

**The readme file of the source code of netGS (network-based genomic selection)**

# 1. System Requirements

## 1.1 Hardware requirements

All codes require only a standard computer with enough RAM to support the in-memory operations.

## 1.2 Software requirements

### 1.2.1 Operation System (OS) Requirements

All codes can be run under Linux or Windows OS. The code here has been tested on Ubuntu 14.04 LTS.

### 1.2.2 Programming software

### R language and Matlab are needed to run all codes. The code here has been tested on R (version 3.4.4) and Matlab (version 8.5 (R2015a)).

# 2. Installation Guide

All installation guide present below is only for *Linux OS*. For *Windows OS*, it might be slightly different.

2.1 R language

### R language can be installed automatically under *Linux OS* using the demand below:

*sudo apt-get install r-base*

In order to install R, you need the password of *sudo* account. The installation could take few minutes on a normal desktop computer.

After successful install R, you could simply type '*R'* in the terminal to open a workspace under R, then run the code written in R language.

The netGS depends on two R packages: rrBLUP and R.matlab, which can be installed using the commands below in R workspace:

*install.packages(rrBLUP)*

*install.packages(R.matlab)*

2.2 Matlab

Matlab can be installed follow the instruction in graphical user interface (GUI) after download Matlab installation compress file. A license is needed to install Matlab. The installation could take up to half an hour on a normal desktop computer.

After successful install Matlab, you could simply type '*matlab'* in the terminal to open a workspace under Matlab, then run the code written in Matlab language.

The netGS depends on Matlab toolbox cobra, solver glpk and cvx.

To install cobra toolbox, you can clone the repository in the terminal using:

*git clone --depth=1 https://github.com/opencobra/cobratoolbox.git cobratoolbox*

then change to the folder *cobratoolbox/* and run the command below in Matlab:

*initCobraToolbox*

To install the solver glpk in Matlab, simply download *glpkcc.mex** files and unzip it, then add the path where you put glpkmex to Matlab.

To install the solver cvx in Matlab, similarly, download the package and unzip it, then add the path where you put cvx to Matlab, and run the below command in Matlab:

*cvx_setup*

**3. Example**

netGS needs the following initial data (the corresponding file name in brackets):

- Metabolic network (model.xml)

The metabolic network should be in the format that can be read by Cobra, including the information at least: stoichiometric matrix (S), gene-protein-reaction rules (rules), lower and upper bounds (lb and ub).

- Phenotypic data (biomass_optN.csv or biomass_optNlowN.csv)

The rows correspond to different genotypes and columns correspond to measured biomass in the given environments.

- Genotypic data (snp.csv)

The rows correspond to SNPs and columns correspond to different genotypes. '1' denotes as the major alleles and '-1' denotes as the minor alleles.

- Population structure data (pop_pca.csv)

The columns correspond to principle components (PCs) calculated from genome SNP matrix. Top ten PCs included in this example.

- Genotype-specific biomass reaction (biomreaction.csv)

The rows correspond to metabolites of biomass reaction in stoichiometric matrix and columns correspond to different genotypes.

- Cross-validation folds (foldid.csv)

The rows correspond to different genotypes and columns correspond to cross-validations. The value denotes which fold each genotype belongs to in a random scenario.

The example showed here is an Arabidopsis genome-scale metabolic model with 407 metabolites and 549 reactions. Phenotypic data is the measured fresh weight (i.e. biomass) in 67 Arabidopsis accessions, and genotypic data is 1,824 enzymatic SNPs. Genotype-specific biomass reactions are calculated by the 30 measured soluble metabolites as well as starch for every genotype in corresponding environments.

Noted that the phenotypic data used in netGS within environment is normalized, and not normalized when prediction cross environments in the example data.

**4. Instructions**

4.1 netGS

All codes and data are under the folder named ‘netGS’.

Because the codes were written both in Matlab and R, four separate files are presented followed the steps in netGS. Please change the path name in the first section of the code to the path on your computer.

- FluxDist.m

This code should be run in Matlab.

This code is used to estimate the reference flux distribution of Col-0 based on flux balance analysis (FBA) and other genotypes based on minimization of quadratic program.

The output including: the reference flux distribution both in *.mat* format (fluxcol0.mat) and *.csv* format (fluxcol0.csv), the other genotype flux distribution both in *.mat* format (fluxgenotype.mat) and *.csv* format (fluxgenotype.csv), and the index for nonzero flux in reference flux distribution (nonzeroid.csv).

The code could take few minutes for the example on a normal desktop computer.

- FluxDist_pFBA.m

This code should be run in Matlab.

This code is used to estimate the reference flux distribution of Col-0 based on pFBA and other genotypes based on minimization of quadratic program.

The output including: the reference flux distribution both in *.mat* format (fluxcol0_pFBA.mat) and *.csv* format (fluxcol0_pFBA.csv), the other genotype flux distribution both in *.mat* format (fluxgenotype_pFBA.mat) and *.csv* format (fluxgenotype_pFBA.csv), and the index for nonzero flux in reference flux distribution (nonzeroid_pFBA.csv).

The code could take few minutes for the example on a normal desktop computer.

- BayesC.R

This code should be run in R.

This code is used to perform the classical GS via BayesC for each non-zero fluxes and estimate the prediction accuracy.

The output including: the predicted flux value for each replicate in three-fold cross validation (fluxpredict_ BayesC _*.csv) and the prediction accuracy of each flux in each cross validation (fluxpredict_ BayesC _cor.csv).

The code could take up to hours for the example on a normal desktop computer.

- BayesC _pop.R

This code should be run in R.

This code is used to perform the classical GS via BayesC with population structure for each non-zero fluxes and estimate the prediction accuracy.

The output including: the predicted flux value for each replicate in three-fold cross validation (fluxpredict_ BayesC _pop_*.csv) and the prediction accuracy of each flux in each cross validation (fluxpredict_ BayesC _pop_cor.csv).

The code could take up to hours for the example on a normal desktop computer.

- rrBLUP.R

This code should be run in R.

This code is used to perform the classical GS via rrBLUP for each non-zero fluxes and estimate the prediction accuracy.

The output including: the predicted flux value for each replicate in three-fold cross validation (fluxpredict_rrBLUP_*.csv) and the prediction accuracy of each flux in each cross validation (fluxpredict_rrBLUP_cor.csv).

The code could take up to half an hour for the example on a normal desktop computer.

- rrBLUP_pop.R

This code should be run in R.

This code is used to perform the classical GS via rrBLUP with population structure for each non-zero fluxes and estimate the prediction accuracy.

The output including: the predicted flux value for each replicate in three-fold cross validation (fluxpredict_rrBLUP_pop_*.csv) and the prediction accuracy of each flux in each cross validation (fluxpredict_rrBLUP_pop_cor.csv).

The code could take up to half an hour for the example on a normal desktop computer.

- Biomass.m

This code should be run in Matlab.

This code is used to estimate the genotype flux distribution in steady-state by minimization of quadratic program. The biomass flux included in this flux distribution is used as the final biomass prediction of netGS.

The output including: the final flux distribution for each replicate and each fold in cross validations (biomasspredict_r*_f*.csv) and the number of fluxes included above the threshold (numfluxbyr2.csv).

The code could take up to an hour for the example on a normal desktop computer.

- Correlation.R

This code should be run in R.

This code is used to check the correlation coefficient between predicted biomass in netGS and measured biomass as the netGS prediction accuracy.

The output including: the correlation coefficient for each replicate and each fold in cross validations (biomcorr.csv).

The code could take few seconds for the example on a normal desktop computer.

4.2 netGS across environments

All codes and data are under the folder named ‘netGS_env’.

Please change the path name in the first section of the code to the path on your computer. If you want to use a new folder, please make sure the results of rrBLUP in previous section (fluxpredict_*.csv and fluxprediction_cor.csv) are copied here or indicate the path to access these results.

- FluxDist.m

This code should be run in Matlab.

This code is used to estimate the reference flux distribution of Col-0 in two environments (optimal and low N) based on FBA and minimization of quadratic program.

The output including: the reference flux distribution in optimal N condition both in *.mat* format (fluxcol0_optN.mat) and *.csv* format (fluxcol0_optN.csv), the reference flux distribution in low N condition both in *.mat* format (fluxcol0_lowN.mat) and *.csv* format (fluxcol0_lowN.csv), and the index for nonzero flux in reference flux distribution in optimal N (nonzeroid.csv).

The code could take few seconds for the example on a normal desktop computer.

- Biomass.m

This code should be run in Matlab.

This code is used to estimate the genotype flux distribution in steady-state in low N condition by minimization of quadratic program. The biomass flux included in this flux distribution is used as the final biomass prediction in low N condition.

The output including: the final flux distribution for each replicate and each fold in cross validations (biomasspredict_lowN_r*_f*.csv).

The code could take up to an hour for the example on a normal desktop computer.

- Correlation.R

This code should be run in R.

This code is used to check the correlation coefficient between predicted biomass in low N condition in netGS and measured biomass in low N condition as the netGS prediction accuracy across environments.

The output including: the correlation coefficient for each replicate and each fold in cross validations (biomcorr_lowN.csv).

The code could take few seconds for the example on a normal desktop computer.

4.3 netGS robustness

All codes and data are under the folder named ‘netGS_robust’.

Please change the path name in the first section of the code to the path on your computer.

- FluxDist.m

This code should be run in Matlab.

This code is used to test the robustness of flux distribution of Col-0 based on FBA and minimization of quadratic program.

The output including: the reference flux distribution both in *.mat* format (fluxcol0.mat) and *.csv* format (fluxcol0.csv), the random sampled reference flux distribution both in *.mat* format (fluxcol0_sample.mat) and *.csv* format (fluxcol0_sample.csv), the random sampled reference flux distribution in steady-state as robustness in both in *.mat* format (fluxcol0_robust.mat) and *.csv* format (fluxcol0_robust.csv), and the index for nonzero flux in reference flux distribution (nonzeroid.csv).

The code could take few minutes for the example on a normal desktop computer.

4.4 For your data

These code can be simply used for any other dataset with the same format of the initial files showed above. You could modified the number of genotypes, number of fluxes in your model and the number of cross-validation replications.

**5. Reference**

Tong, H., Küken, A. & Nikoloski, Z. Integrating molecular markers into metabolic models improves genomic selection for growth. (Under submission)

Please see the Methods section in this paper for the model details in mathematical equations.

Any further questions: tong@mpimp-golm.mpg.de
