# Supplementary material for: Integrating molecular markers into metabolic models improves genomic selection for Arabidopsis growth
Source: Nat Commun. 2020 May 15;11:2410. doi: 10.1038/s41467-020-16279-5 (PMC7229213; doi:10.1038/s41467-020-16279-5)
Supplement: Supplementary file 2 — Peer Review File [file 41467_2020_16279_MOESM2_ESM.pdf]

Reviewers' comments:

Reviewer #1 (Remarks to the Author):

The current work presented a modeling approach for predicting the genomic selection (GS) via a case study of the growth prediction of Arabidopsis thaliana based on accession-specific metabolic models. The authors claimed that the approach outperforms the prediction accuracy of growth within and across nitrogen environments, and can be cost-effectively applied to crop growth as an agronomically relevant trait unlike conventional GS techniques relying on large-scale phenotypic datasets under multiple environments. Although the current work is interesting, its applicability to crop development is still questionable since there is big gap between Arabidopsis thaliana and crop like rice, wheat and corn in terms of genome content, size and complexity. Another critical issue is the inherent limitation of internal flux quantification due to the nature of underdetermined system, i.e., existence of multiple solutions so it is very risky to talk about internal flux values and corresponding metabolic utilization which may mislead the results. So, its applicability can be only demonstrated via crop case study, e.g., rice, employing unbiased FBA approach, and experimental validation should be conducted to justify it.

Reviewer #2 (Remarks to the Author):

In this manuscript, the authors apply metabolic models to improve the accuracy of phenotype prediction from genome variation in plant species over classical statistical approaches. This is an extremely clever, and to my knowledge, novel use of metabolic models. As the authors say, models offer a mechanism for injecting metabolic pathways and gene association knowledge into the otherwise completely correlation based GWAS style techniques for associating genotype and phenotype. The method appears quite sounds although some aspects of the method could be clarified. I raise these concerns below. The results are compelling, indicating the value of using this metabolic knowledge to aid in phenotype prediction from genotype. Overall, the method, the models, and the new data released in this paper will be a fantastic resource to the academic community.

Comments:
1.) One of the primary terms in the biomass equation that influences biomass yield prediction in metabolic models is the ATP hydrolysis term that reflects the energy consumed by biomass synthesis. Typically this parameter is adjusted to match measured yields. What value was used for the ATP hydrolysis term in the reference and various accession strains? If the ATP term coefficient was varied in the accessions, how did the authors determine these various values? This isn’t mentioned in the methods.

2.) In the methods, the authors mention that the measured biomass values were scaled to fit the models. Specifically: "the maximum biomass of the model under the loosest constraints was considered larger than the measured biomass”. This sentence isn’t entirely clear in my reading, and this seems to be a critical detail. Maybe an example or equations could be provided?

3.) The authors say: "The flux of each reaction Ri is modeled according to GS based on the given set of SNPs.”. I understand this to mean that the authors constructed a function for each reaction such that the flux can be computed (as closely as possible to the computed fluxes for the training strains) based on the SNPs present in the genes associated with the reaction. How accurate was this fitting to the training set? Were there reactions that fit particularly poorly? How many nonzero reactions had no gene associations and thus could not be addressed by this analysis? Were ALL SNPs used in this fitting process, only all enzymatic SNPs, or just the SNPs falling within the genes associated with each reaction? If the authors only used one of these options, did they explore using some of the other options when deriving the method? It sounds from the results that all SNPs and all enzymatic SNPs were both tried, but it’s not totally clear. Were different SNPs important when computing different flux

values? How flexible was this computation (could equally good results be obtained with different SNPs or SNP weights included in the predictive function gi().

4.) I'm also uncertain how this approach is dealing with the very significant tissue-level variation in plant metabolism. Regarding the biomass quantification that was done on 67 strains, was there a particular tissue that was assessed, or was the whole plant homogenized. What tissue were the starch and carboxylation ratios measured in? Are we sure those values are consistent for all tissues? Overall, do the authors think tissue level variation in metabolism is a concern? It seems this should be minimally discussed in the manuscript.

Reviewer #3 (Remarks to the Author):

The study by Tong, Kuken and Nikoloski describes an approach for integrating metabolic models within Genomic Selection (GS). The work done is interesting and it bridges the fields of metabolic pathways and physiology with the more statistically-oriented literature on GS.

Main comments

1. Several authors have considered integrating physiological knowledge into genomic models in the past (the literature goes back to QTL mapping, and has more recently integrated into GS). Most of the studies appeared under the umbrella of 'Crop Models'. I am not on expert on this field but I do suggest the authors to review this literature. I suggest starting from the studies lead by Mark Cooper (e.g., https://www.ncbi.nlm.nih.gov/pubmed/26121133 and references thereof). The authors should: (1.1) put their research in the context of the literature on crop models and (2.2) Identify more clearly what is novel compared with work previously published such as the one referenced above.

2. Was prediction accuracy quantified in a correct way? It seems from the description of the analyses that the authors first used the entire data set to conduct flux-balance analyses. This was used to asses 549 reactions and the results were used to select reactions that had non-zero fulxes, to asses variability of the reactions, and to obtain flux profiles for every genotype (accession). Then, these models that were derived using the entire data set were integrated with GS models in a 3-fold CV to assess accuracy. If my understanding of the methodology is correct, the prediction accuracies reported for netGS are likely to be upwardly-biased. Unbiased estimation of prediction accuracy requires that all the steps involved in the development of a model are applied to the training data only. When this is not respected, the prediction accuracies are often upwardly bias and this may explain to some extent the superiority of the netGS over traditional GS (traditional GS was entirely trained within the 3-fold CV, but some steps of the training of the netGS seemed to have done in the entire data set before the 3-Fold CV was implemented). The problem above described is well known in the statistical and machine learning literature as well as in QTL mapping and genomic prediction (e.g., Beavis effect).

3. The authors emphasize the importance of using accession-specific 'models'. The use of accession-specific models trained on the entire data set would be another reason that may lead to upwardly biased estimates of accuracies. In addition to that, I would like to highlight that if one would need to estimate genotype-specific metabolic parameters/models, that would make the implementation of netGS very costly and potentially ineffective because it would require to collect data on each candidate of selection to tune up the genotype-specific flux models before these candidates can be selected. This would lead to a very long generation interval and thus slow genetic progress compared to what could do with rapid cycling (i.e. rapid rounds of GS without phenotyping).

Minor (yet important) suggestions:

4. I suggest to assess population structure (via PC analyses of genotypes) and include among the models you compare against a PC model based on, say 5 -to-10 pc. Such an analyses would quantify how much of the prediction accuracy obtained with GS and netGS could be attributed to population structure.

5. I also suggest to considering Bayesian models as a reference (e.g., BayesB, BayesC, Bayesian Lasso).]]

6. The sample size is a clear limitation of the study; however, I understand that large data sets with the type of information needed for this study do not abound. I suggest to: (6.1) perform formal statistical analyses to assess whether the correlations were significantly different (the error bars on the correlations are, as expected for the sample size, rather large and the correlations do not seem to be significantly different) and to (6.2) replicate the 3 fold CV at least 30 times (again, in each fold you would need to retrain the models from zero, including all the steps, in the training folds and then apply the trained model to the left-out fold).

7. I also believe that the across environment analyses presented towards the end may suffer from the Winner's curse because there seems to be a step where some aspects of the netGS model were decided using information from the entire data set.

Response to editor and reviewers' comments to

'Integrating molecular markers into metabolic models outperforms genomic selection'

Reviewer #1:

"The current work presented a modeling approach for predicting the genomic selection (GS) via a case study of the growth prediction of Arabidopsis thaliana based on accession-specific metabolic models. The authors claimed that the approach outperforms the prediction accuracy of growth within and across nitrogen environments, and can be cost-effectively applied to crop growth as an agronomically relevant trait unlike conventional GS techniques relying on large-scale phenotypic datasets under multiple environments."

Response: We thank the reviewer for summarizing the main results from our study. We would like to stress that our results are supported by extensive robustness checks and comprehensive comparative analyses against contending approaches for genomic selection, as detailed in the remainder of our response below as well as in the comments of the other two reviewers. Therefore, we are convinced that this work is a valuable pioneering contribution that opens a new branch for rapid crop selection and breeding.

"Although the current work is interesting, its applicability to crop development is still questionable since there is big gap between Arabidopsis thaliana and crop like rice, wheat and corn in terms of genome content, size and complexity."

Response: The proposed netGS approach is a first that integrated ideas from genomic selection with metabolic modeling. Thorough testing of the netGS approach requires well-understood model plant for which there are sufficient data on growth, metabolic measurements from dedicated experiments under different environments assessed with the same metabolomics technology (to minimize noise from technical procedures), availability of genomics data (e.g. SNPs) along with high-quality metabolic networks. These requirements are fulfilled for *Arabidopsis thaliana*, and we use our study as a proof-of-principle to demonstrate the benefits of integrating metabolic modeling with the existing, purely statistical, approaches for genomic selection. Since netGS is a constraint-based approach, it can readily be applied to crops, despite their different complexity in comparison to *Arabidopsis*. The reason is that netGS requires high-quality metabolic models which already exist for crops, e.g. rice[1] and maize[2], for which there are also ample genomics data resources available[3,4]. However, there are still no dedicated experiments in which growth and quantitative metabolomics data are measured under different environments to obtain accession-specific models. While combining data from different studies is one possibility to address this point, such an approach may lead to artefacts due to differences in growth conditions or the usage of different metabolomics technologies between different labs—a point which in our view precludes

unbiased comparison. The point that *A. thaliana* is an adequate system for a proof-of-concept study is recognized by the other reviewers, who support our view that such dedicated experiments are needed to deploy netGS to agronomically relevant crops.

"Another critical issue is the inherent limitation of internal flux quantification due to the nature of underdetermined system, i.e., existence of multiple solutions so it is very risky to talk about internal flux values and corresponding metabolic utilization which may mislead the results. So, its applicability can be only demonstrated via crop case study, e.g., rice, employing unbiased FBA approach, and experimental validation should be conducted to justify it."

Response: As pointed by the reviewer, any constraint-based approach inherently includes solutions from an underdetermined linear system (due to the nature of the metabolic network that contains more reactions than metabolites). The consideration of different constraints is what narrows down the solution space under optimization of particular objectives. In our study, we use the constraint from ratio of measured fresh weight—a proxy for growth—to constrain the ratio of fluxes through the biomass reaction from *accession-specific* metabolic models (obtained from consideration of quantitative metabolomics data) under the assumption that an accession minimizes its flux distribution with respect to that of the reference (i.e. Col-0). Moreover, we include physiological constraints about the ratio of fluxes through the RuBisCO-catalyzed reactions as well as the ratio between fluxes through starch and sucrose synthesis. In netGS we first determine a flux distribution for the reference, which is then used to obtain the flux distribution for the remaining accessions. In other constraint-based approaches, one usually selects only one of the optimal solutions, which is then used for subsequent analysis. In contrast, already in the original version of our manuscript, we already analyzed the robustness of the predictions by selecting other optimal solutions or showing that the variability in majority of the fluxes under the abovementioned constraints is negligible and does not affect our predictions. This was done both with respect to the reference flux distribution as well as the distributions of the remaining accessions. In addition, we used maximal enzyme activities to validate that the predicted flux distributions are in line with these additional data sets. In all cases, we demonstrated that our netGS approach is unbiased, thus fully addressing the reviewer's comment.

Reviewer #2:

"In this manuscript, the authors apply metabolic models to improve the accuracy of phenotype prediction from genome variation in plant species over classical statistical approaches. This is an extremely clever, and to my knowledge, novel use of metabolic models. As the authors say, models offer a mechanism for injecting metabolic pathways and gene association knowledge into the otherwise completely correlation based GWAS style techniques for associating genotype and phenotype. The method appears quite sounds although some aspects of the method could be clarified. I raise these concerns below. The results are compelling, indicating

the value of using this metabolic knowledge to aid in phenotype prediction from genotype. Overall, the method, the models, and the new data released in this paper will be a fantastic resource to the academic community."

Response: We thank the reviewer for the positive assessment of the main points of our manuscript. This is the first attempt combining the genome-scale metabolic network and classical statistical models for plant performance prediction. We believe that the proposed netGS approach will open a new era of the plant phenotypic predictions and further help crop selection and breeding processes.

Comments:

"1.) One of the primary terms in the biomass equation that influences biomass yield prediction in metabolic models is the ATP hydrolysis term that reflects the energy consumed by biomass synthesis. Typically this parameter is adjusted to match measured yields. What value was used for the ATP hydrolysis term in the reference and various accession strains? If the ATP term coefficient was varied in the accessions, how did the authors determine these various values? This isn't mentioned in the methods."

Response: We agree with the reviewer that the contribution of ATP in the biomass reaction has an effect on the predicted growth. However, we only had access to accession-specific quantitative metabolomics measurements for 30 components of the biomass reactions, including: free amino acids, sugars, TCA-related metabolites and starch (as pointed out on pp. 19 of the original version of the manuscript). These measurements did not include accessions-specific ATP, as can be seen by the same coefficient in the biomass reactions included in Supplementary Table 1. We indicate that it is the constraint on the ratio between the growth of two accessions which accounts for the difference in the ATP usage, as indicated in the resulting flux distributions. Indeed, we find that the ATP hydrolysis reaction (as ATPase reaction in Supplementary Table 7) varied from 100.27 to 107.13 with the coefficient variance (CV) of 1.05, and we observed a relatively high correlation (R^2=0.78) between the flux through the ATP hydrolysis reaction and biomass, in support of our claim and in line with the reviewer's comment. We mention these points explicitly in the updated version of the methods.

"2.) In the methods, the authors mention that the measured biomass values were scaled to fit the models. Specifically: "the maximum biomass of the model under the loosest constraints was considered larger than the measured biomass". This sentence isn't entirely clear in my reading, and this seems to be a critical detail. Maybe an example or equations could be provided?"

Response: We thank the reviewer for raising this important point. In the following, we provide all details and reasoning for the approach:

Maximizing the flux through the biomass reaction for a constraint-based model has a unique optimum. To ensure the feasibility of the optimization program, measured biomass values were scaled to fit the range of biomass values that can be obtained with the used model. To this end, for each genotype we first determined the maximum flux through the genotype-

specific biomass reaction under the following constraints: (*i*) steady state, (*ii*) bounds on the ratio between the carboxylation and oxygenation reactions and between starch and sucrose synthesis, (*iii*) non-negative carboxylation flux, for biological meaningfulness, and (*iv*) genotype-specific biomass reaction. This leads to the following linear program:

$$\max_{v^Z} \; v^Z_{biomass}$$

s.t.

$$N \cdot v^Z = 0,$$

$$\forall i, 1 \leq i \leq n, \alpha_i \leq v^Z_i \leq \beta_i,$$

$$0.94 v^Z_{oxygenation} \leq v^Z_{carboxylation} \leq 3.81 v^Z_{oxygenation},$$

$$0.79 v^Z_{sucrosesynth} \leq v^Z_{starchsynth} \leq 3.37 v^Z_{sucrosesynth},$$

$$v^Z_{carboxylation} \geq 0,$$

$$N_{biomass} \leftarrow N^Z_{biomass}.$$

We used the average of the maxima, $v^{Z,max}_{biomass}$, from genotype-specific models to define the *model scaling parameter* $s_{model} = \overline{v^{Z,max}_{biomass}}$. To determine the genotype-specific flux distribution by the quadratic program P2 in the manuscript, we imposed the constraint that $v^{Z,ratio}_{biomass} = \frac{M_Z}{M_{Col0}} v^{Col0}_{biomass}$, where M_{Col0} and M_Z are the measured fresh weights of Col-0 and genotype Z, respectively, and $v^{Col0}_{biomass}$ is the biomass flux in the estimated reference flux distribution. Then, the measured maximum biomass was defined as the maximum value among all genotypes, $s_{measurement} = max(v^{all,ratio}_{biomass})$, and is referred to as the *measurement scale parameter*. To ensure the feasibility of our approach, the biomass flux for the genotype Z in the optimization program was scaled by the two scaling parameters above, as $v^{Z,scale}_{biomass} = \frac{(s_{model} - \delta)}{s_{measurement}} v^{Z,ratio}_{biomass}$, where δ is a tunable parameter, here set of the value of 1.1×10^{-4}.

The related part was revised in the manuscript with the detailed programs and parameter values used.

"3.) The authors say: "The flux of each reaction Ri is modeled according to GS based on the given set of SNPs.". I understand this to mean that the authors constructed a function for each reaction such that the flux can be computed (as closely as possible to the computed fluxes for the training strains) based on the SNPs present in the genes associated with the reaction. How accurate was this fitting to the training set? Were there reactions that fit particularly poorly? How many nonzero reactions had no gene associations and thus could not be addressed by this analysis? Were ALL SNPs used in this fitting process, only all enzymatic SNPs, or just the SNPs falling within the genes associated with each reaction? If the authors only used one of these options, did they explore using some of the other options when deriving the method? It sounds from the results that all SNPs and all enzymatic SNPs were both tried, but it's not totally clear. Were different SNPs important when computing different flux values? How

flexible was this computation (could equally good results be obtained with different SNPs or SNP weights included in the predictive function gi().”

Response: We thank the reviewer for these questions, which we believe these have addressed in the original version of the manuscript but they may not have been clearly presented. We provide detailed answers here and streamline the main text accordingly: In this manuscript, we *do not* model the reaction flux based only on the SNPs in the genes associated with the reaction through the gene-protein-reaction rules. The reason is that not all reactions are associated genes, and genomic selection uses usually all available SNPs. Instead, we use two SNP data sets in the analysis: genome-wide SNPs and enzymatic SNPs. The genome-wide SNPs include *all SNPs* available for the population, while the enzymatic SNPs comprise *all SNPs which are on the genes included in the model*. These two sets provide a compromise between all available genomics data and genomics data associated with a gene function. In addition, this avoids the aforementioned issue of neglecting reactions which are not associated any genes. All SNPs in each data set were treated equally, no weight was applied in the rrBLUP models for genomic selection.

We find that the mean prediction accuracy based on genomic selection with 150 cross-validations ranges from -0.204 to 0.461, with an average value of 0.225, for the enzymatic SNPs and from -0.062 to 0.464, with an average value of 0.339, based on the genome-wide SNPs (see Supplementary Table 8). The average accuracy of prediction of fluxes increases by 1.5-fold between the genome-wide and enzymatic SNPs. These findings are also illustrated in Supplementary Figure 6, in which we have highlighted the prediction accuracy for particular reactions. For instance, the reaction with relatively low accuracy is Tre phosphatase, and the reaction with relatively high accuracy is SCA dehydrogenase. The importance of individual SNPs is not relevant in the context of genomic selection, as it is used to determine predictions of good or improved accuracy, rather than to identify putatively causal loci. Interestingly, and surprisingly, we find that the mean accuracy of netGS using enzymatic SNPs and genome-wide SNPs are comparable (i.e. 0.324 and 0.352 in optimal N condition, and 0.199 and 0.266 from optimal N to low N condition, respectively), although there are ~100-fold more SNPs in the genome-wide in comparison to the enzymatic sets (1,824 enzymatic SNPs and 180,859 genome-wide SNPs). The reason for this is that enzymatic SNPs already capture substantial variance of that present in the genome-wide SNPs.

“4.) I’m also uncertain how this approach is dealing with the very significant tissue-level variation in plant metabolism. Regarding the biomass quantification that was done on 67 strains, was there a particular tissue that was assessed, or was the whole plant homogenized. What tissue were the starch and carboxylation ratios measured in? Are we sure those values are consistent for all tissues? Overall, do the authors think tissue level variation in metabolism is a concern? It seems this should be minimally discussed in the manuscript.”

Response: We appreciate this important comment. The metabolomics measurements are obtained from an entire *Arabidopsis* rosette, which were used to estimate fluxes in a typical *Arabidopsis* illuminated leaf, include carboxylation and oxygenation reaction[5]. These were used to parameterize the AraCore model for Col-0 accession[6]. In addition, the fresh weight

used to estimate growth is that of the rosette. We do not model root, stem or other tissues[7,8], but these can be considered in future modeling attempts. To this end, we extended the discussion to account for the important point.

Reviewer #3:

"The study by Tong, Kuken and Nikoloski describes an approach for integrating metabolic models within Genomic Selection (GS). The work done is interesting and it bridges the fields of metabolic pathways and physiology with the more statistically-oriented literature on GS."

Response: We thank the reviewer for the positive assessment of the main points of our manuscript. This is the first attempt combining the genome-scale metabolic network and classical statistical models for plant performance prediction. We believe that the proposed netGS approach will open a new era of the plant phenotypic predictions and further help crop selection and breeding processes.

Main comments

"1. Several authors have considered integrating physiological knowledge into genomic models in the past (the literature goes back to QTL mapping, and has more recently integrated into GS). Most of the studies appeared under the umbrella of 'Crop Models'. I am not on expert on this field but I do suggest the authors to review this literature. I suggest starting from the studies lead by Mark Cooper (e.g., https://www.ncbi.nlm.nih.gov/pubmed/26121133 and references thereof). The authors should: (1.1) put their research in the context of the literature on crop models and (2.2) Identify more clearly what is novel compared with work previously published such as the one referenced above."

Response: Crop growth models (CGMs) are statistical models in which one agronomically relevant plant trait (e.g. yield, biomass) is expressed as a function of factors such as: physiological traits, their underlying QTL or genome-wide markers (SNPs), as well as environmental variables (e.g. temperature, irrigation, solar radiation and soil nutrients). The usage of SNPs in CGMs is referred to as whole genome prediction (WGP). CGMs are particularly used to investigate the effect of environment or genotype-by-environment (G-by-E) effect since environment parameters are directly included in the model. However, the interactions (either G-by-E or epistasis) in CGMs using linear or non-linear regression models are usually two-way interactions, and the computational complexity for parameterizing such models with genome-wide SNPs is still challenging due to the large number of resulting variables. To account for the latter issue, the manuscript referred to in the comments of the reviewer expresses the physiological parameters in terms of genome-wide SNPs by using the Approximate Bayesian Computation (ABC) framework and is based on simulated data for individual genotypes (for which no measurement of physiological parameters are collected). In that sense, a CGM-WGP can be regarded as a two-step approach: (1) regression describing the relationship between physiological parameters and agronomically relevant traits, which is obtained by usage of all or subset of the data (depending on the approach for building the model), and (2) model for the physiological parameters based on the SNPs for unseen

genotypes.

In contrast to CGM, our netGS approach does not include environmental variables in the modeling. The effects of the latter are indirectly considered via the usage of metabolomics profiles. In addition, we use metabolic network models to predict intermediate traits—reaction fluxes—for an individual accession, *independently of all other* accessions (i.e. we do not simulate them based on the ABC framework using SNPs and knowledge of the ranges of the physiological parameters over all genotypes). We achieve this by usage of constraint-based modeling in which we solve a series of optimization problems, as detailed in the methods, which is another principle novelty. These differences are highlighted in the discussion of the updated version of the manuscript.

"2. Was prediction accuracy quantified in a correct way? It seems from the description of the analyses that the authors first used the entire data set to conduct flux-balance analyses. This was used to asses 549 reactions and the results were used to select reactions that had non-zero fluxes, to asses variability of the reactions, and to obtain flux profiles for every genotype (accession). Then, these models that were derived using the entire data set were integrated with GS models in a 3-fold CV to assess accuracy. If my understanding of the methodology is correct, the prediction accuracies reported for netGS are likely to be upwardly-biased. Unbiased estimation of prediction accuracy requires that all the steps involved in the development of a model are applied to the training data only. When this is not respected, the prediction accuracies are often upwardly bias and this may explain to some extent the superiority of the netGS over traditional GS (traditional GS was entirely trained within the 3-fold CV, but some steps of the training of the netGS seemed to have done in the entire data set before the 3-Fold CV was implemented). The problem above described is well known in the statistical and machine learning literature as well as in QTL mapping and genomic prediction (e.g., Beavis effect)."

Response: We understand the reviewer's point and stress that we have not used the entire data set, over all accessions, in the estimation of fluxes (unlike what is done in the paper referred in the comment above). More specifically, there are three key steps in netGS: (*i*) estimation of an accession-specific flux distribution for each accession separately. This is achieved by solving a quadratic program presented in method section where we obtain the flux distribution of a genotype Z. (*ii*) prediction of a reaction flux using SNPs based on rrBLUP. This was carried out by 3-fold cross-validation with 50 repetitions, in which the accessions in the training set are generated randomly and independently in every cross-validation. (*iii*) the steady-state flux distribution in the training set closest to the models of reaction fluxes from step (*ii*) above was obtained, again, for each accession separately without using any additional information from other network models of accessions in the training set. Because the quadratic programs in the first and the third steps are optimized for each accession separately, the training set and testing sets are independent. Therefore, we do not recognize any potential for upward-bias in the prediction of netGS. The related parts of the manuscript have been revised to clarify any misunderstandings.

“3. The authors emphasize the importance of using accession-specific ‘models’. The use of accession-specific models trained on the entire data set would be another reason that may lead to upwardly biased estimates of accuracies. In addition to that, I would like to highlight that if one would need to estimate genotype-specific metabolic parameters/models, that would make the implementation of netGS very costly and potentially ineffective because it would require to collect data on each candidate of selection to tune up the genotype-specific flux models before these candidates can be selected. This would lead to a very long generation interval and thus slow genetic progress compared to what could do with rapid cycling (i.e. rapid rounds of GS without phenotyping).”

Response: We would like to thank the reviewer for raising this important point. A metabolic model for a given accession is obtained by using data only for that accession, via updating the coefficients of the biomass reaction (Supplementary Table 1) with the available quantitative metabolomics data. Therefore, contrary to the statement of the reviewer, we do not use all data to arrive at accession-specific models. In line with the answer to the comment 2, above, we would like to stress that we do not use the entire data set for training, as the optimization programs estimating accession-specific fluxes from the accessions-specific models were solved for each accession separately. Another benefit of netGS is that we do not require accession-specific models for every accession in every environment of interest. Indeed, we show that it suffices to have all accession-specific models for one conditions (in our case, optimal N) and a model for the reference accession in another, second condition (in our case Col-0, under low N) to make predictions for all other accessions in absence of models for the second condition. Phenotyping all accessions in a single condition to measure 30 metabolites can be readily achieved with targeted metabolomics at a low cost, given the generalizability of the models achieved that lead to 50% increase in prediction accuracy over the classical genomic selection. As another alternative, one can explore the usage of genomic selection for the metabolic traits to parameterize the biomass reaction to arrive at accession-specific models for unseen accessions. This alternative is critically presented in the discussion of the updated version of the manuscript as venues to future fruitful research.

Minor (yet important) suggestions:

“4. I suggest to assess population structure (via PC analyses of genotypes) and include among the models you compare against a PC model based on, say 5 -to-10 pc. Such an analyses would quantify how much of the prediction accuracy obtained with GS and netGS could be attributed to population structure.”

Response: We have included the model in which we considered the inclusion of the first ten principle components (PCs) from the genome-wide SNP matrix to check the population structure effect on the GS predictions. The results are presented in Supplementary Table 16 and 17 for predictions within optimal N condition and from optimal N to low N condition, respectively. Three approaches were employed: rrBLUP model using genome-wide SNPs, rrBLUP model using enzymatic SNPs, and BayesC model using enzymatic SNPs. The BayesC based on genome-wide SNPs was not included due to the substantial computational time required. Nevertheless, based on the observation from the rrBLUP models, the performance

for the two SNP sets should be quite similar. Four types of models were obtained in each approach: GS (rrBLUP or BayesC) for fresh weight, netGS without accession-specific biomass reaction, netGS with accession-specific biomass reaction, and robustness test by random sampling within a specified relative error used for comparison. We found: (1) an increase in predicting accuracy based on the enzymatic SNPs and the first ten PCs, and (2) nearly no increase using the genome-wide SNPs and the first then PCs with the rrBLUP approach. These findings demonstrated that the population structure effect helps the growth prediction for the small SNP set; as the number of SNPs increases, the population structure does not have effect on the predictions of growth, since it may already be compensated by the sheer number of SNPs used. However, the BayesC approach is very sensitive to the inclusion of the first ten PCs, and the GS prediction accuracies dropped when including ten continuous vectors to the binary SNP matrix. Most importantly, in all cases, netGS outperformed GS.

"5. I also suggest to considering Bayesian models as a reference (e.g., BayesB, BayesC, Bayesian Lasso)."

Response: We have included the BayesC algorithm for genomic selection as a reference for comparing our netGS approach in the revised version. Similarly, the BayesC approach was applied by using the enzymatic SNPs due to the large computation time for genome-wide SNP set with the same four models, as above. The BayesC models were calculated based on the R package BGLR (Bayesian Generalized Linear Regression)[9]. The results are presented in Supplementary Table 14 and 15 for GS prediction accuracy within optimal N condition and from optimal N to low N condition, respectively. The GS prediction accuracy of BayesC is similar to that of rrBLUP, in line with studies from classical genomic selection. Altogether, we found that the accuracy of netGS increased by 31.6% and 67.8% in comparison to the GS based on BayesC within and between environment, respectively. Therefore, netGS approach outperforms accuracy of prediction within and between environments with BayesC as a reference.

"6. The sample size is a clear limitation of the study; however, I understand that large data sets with the type of information needed for this study do not abound. I suggest to: (6.1) perform formal statistical analyses to assess whether the correlations were significantly different (the error bars on the correlations are, as expected for the sample size, rather large and the correlations do not seem to be significantly different) and to (6.2) replicate the 3 fold CV at least 30 times (again, in each fold you would need to retrain the models from zero, including all the steps, in the training folds and then apply the trained model to the left-out fold)."

Response: We have performed t-tests to assess if the means of the correlation coefficients from GS and netGS differ. Indeed, we found that the difference between the two is significant in both within ($p=6.28\times10^{-6}$) and between environment ($p=2.72\times10^{-6}$) with enzymatic SNPs. Figure 3 has been updated in the manuscript accordingly. We thank the reviewer for this valuable suggestion.

“7. I also believe that the across environment analyses presented towards the end may suffer from the Winner’s curse because there seems to be a step where some aspects of the netGS model were decided using information from the entire data set.”

Response: As stated above, our netGS approach does not rely on using the entire data set in the training for any of the netGS steps. The step the reviewer refers to also considers individual accessions in absence of information and data of other accessions. As explained above, and detailed in the discussion, across environment analyses do not use accession-specific models for the second environment, and thus do not suffer from the Winner’s curse. The related parts of the manuscript are revised to clarify any misunderstandings.

Reference

1. Lakshmanan, M. et al. Unraveling the light-specific metabolic and regulatory signatures of rice through combined in silico modeling and multiomics analysis. *Plant Physiol.* **169**, 3002-3020 (2015).
2. Simons, M. et al. Assessing the metabolic impact of nitrogen availability using a compartmentalized maize leaf genome-scale model. *Plant Physiol.* **166**, 1659-1674 (2014).
3. Chen, W. et al. Genome-wide association analyses provide genetic and biochemical insights into natural variation in rice metabolism. *Nat. Genet.* **46**, 714–721 (2014).
4. Yang, N. et al. Genome wide association studies using a new nonparametric model reveal the genetic architecture of 17 agronomic traits in an enlarged Maize association panel. *Plos Genet.* **10**, e1004573 (2014).
5. Szecowka, M. et al. Metabolic fluxes in an illuminated Arabidopsis rosette. *Plant Cell* **25**, 694-714 (2013).
6. Arnold, A. & Nikoloski, Z. Bottom-up metabolic reconstruction of Arabidopsis and its application to determining the metabolic costs of enzyme production. *Plant Physiol.* **165**, 1380-1391 (2014).
7. de Oliveira Dal'Molin, C. G. et al. A multi-tissue genome-scale metabolic modeling framework for the analysis of whole plant systems. *Front. Plant Sci*. **6**, 4 (2015).
8. Seaver, S. M. D. et al. Improved evidence-based genome-scale metabolic models for maize leaf, embryo, and endosperm. *Front. Plant Sci.* **6**, 142 (2015).
9. Perez, P. & de los Campos, G. Genome-Wide Regression and Prediction with the BGLR Statistical Package. *Genetics* **198**, 483-495 (2014).

Reviewers' comments:

Reviewer #1 (Remarks to the Author):

My two major questions and doubts were not addressed at all.
Experimental verification is highly required to show applicability of the approach. In addition, unbiased FBA such as pFBA or FBA with relevant constraints such as molecular crowding or kinetic information should be conducted. Otherwise, the current theoretical approach may be more relevant to Systems Biology or Bioinformatics journals.

Reviewer #2 (Remarks to the Author):

I appreciate the effort the authors have taken to respond to all of my comments. The manuscript is much clearer now, and I believe all of my questions have been resolved.

Reviewer #3 (Remarks to the Author):

I have only two comments:

(1) I don't think the differences in accuracy (see Fig 3.) are that substantial to justify the title. This is particularly important considering that the difference in accuracy is small and it is based on a single (very small) data set. Will these results replicate in other Arabidopsis data sets. Will these extend to other traits. Will this result hold with larger N and in other species? These are all very important questions that the manuscript cannot answer. For that reason I would suggest a more sober tone in the title (make it more descriptive, e.g. Integrating Metabolic Models into Genomic Selection), abstract, discussion and conclusions.

(2) I am not 100% satisfied with the answer about the cross-validation. My concern is not with genetic accessions but on weather data from testing set was in any steps of the model building. No data from testing sets should be used for model building, otherwise the estimated accuracies will be upwardly bias. By model building I include anything that may involve estimation of flux, flux distribution, feature selection, parameter estimation etc. The model must be entirely build within each of the training sets and then just applied by using only the SNP data from the testing set. Please confirm if this was the case.

Rebuttal Letter to the Reviewer's Comments on

'Integrating molecular markers into metabolic models outperforms genomic selection'

Hao Tong, Anika Küken, Zoran Nikoloski

The Rebuttal Letter includes the original comments from the editor and the three reviewers together with our point-by-point responses including the clarification of the raised points. The original comments are provided in regular font, while our response appear in a bold font. The revised manuscript is submitted along with the letter.

Editor's comments:

You will see that, while the reviewers find your work of interest, they raise substantive concerns on the lacking of demonstration of application and justification of accuracy. Unfortunately, these reservations are sufficiently important to preclude publication of this study in Nature Communications.

In the original and revised versions of the manuscript, we stressed that applications of netGS necessitates access to data from dedicated experiments in which quantitative metabolomics data, necessary for assembly of genotype-specific biomass reactions, have been gathered. To the best of our knowledge, such data sets are currently available only for *Arabidopsis thaliana*. Reviewers #2 and #3 recognized this important point, and accept our argument that netGS, as currently presented, provides a thorough assessment of this pioneering approach for this model plant. Conducting the requested study for a crop would require at least two additional years of measurements and establishing collaborations (e.g. with researchers in rice) to conduct dedicated experiments, which we consider an unjustified request for a computational systems biology study.

As for the accuracy of the predictions, they have not changed from the original submissions and they demonstrate that netGS provides an increase in prediction accuracy for growth within and across nitrogen environments by 32.6% and 51.1%, respectively. In addition, in the revised manuscript we show that netGS leads to an increase in prediction for growth from optimal nitrogen to <u>low carbon environment</u> by 50.4%, thus further demonstrating the applicability of the approach.

We are convinced that these additions further strengthen the claims of our study.

Reviewer #1 (Remarks to the Author):

My two major questions and doubts were not addressed at all.

We strongly disagree with the reviewer's assessment.

netGS is a versatile approach as it is constraint-based and can be applied to both model plants and crops alike. In our response, we emphasized that netGS necessitates quantitative metabolomics data from multiple conditions in a natural diversity panel of genotypes. To the best of our knowledge, such data set is not available neither for rice nor for maize, for which high-quality metabolic models exists (as argued in the main

text as well). Developing such a data set would require at least two more years of experiments and measurements, which we find is an unjustified request for a computational systems biology study.

The other point in the first round of reviews dealt with the existence of multiple solutions in the process of determining flux distributions, and the reviewer suggested (as an example) to use unbiased FBA approach. To the best of our knowledge, there is no such FBA approach, so additional analysis in this direction could not be performed. In fact, we went on to analyze the robustness of the flux distributions both in the reference genotype (Col-0) as well as in the flux distributions of the other genotypes—and demonstrated little to no variability in the fluxes. These findings, therefore, demonstrated that our original approach does not suffer from issue of alternative optima. This is in stark contrast to the claim of the reviewer that we have not addressed her/his comments.

Experimental verification is highly required to show applicability of the approach. In addition, unbiased FBA such as pFBA or FBA with relevant constraints such as molecular crowding or kinetic information should be conducted. Otherwise, the current theoretical approach may be more relevant to Systems Biology or Bioinformatics journals.

We would like to stress that we do show experimental verification of the approach, since we use data from different environment to demonstrate the prediction accuracy—namely, low nitrogen (in the original and revised version) and low carbon, in the version of the rebuttal.

As for the other requests, in the revised version of the manuscript, we also applied pFBA and demonstrated that the performance of netGS is not altered. Although pFBA is not unbiased (namely, it, too, suffers from alternative optima), we demonstrate that pFBA, like FBA with constraints as in our study, show little to no variability on the determined flux distributions, and results in a similar improvement as the initial version of netGS (please, refer to Supplementary Tables 18 and 19 for within and across nitrogen environments).

Finally, the request to perform FBA with other relevant constraint, like molecular crowding or kinetic information, are simply infeasible given the current state of plant metabolic modeling. For instance, performing FBA with molecular crowding requires that we have information on total protein available (or we have access to the crowding coefficient in different environments)—which are not available not only for multiple genotypes, but also not for *A. thaliana* reference genotype Col0. In addition, performing analyses with inclusion of kinetic information (e.g. following the MOMENT approach), requires that we have access to plant-specific k_{cat} data for every reaction in the model. Based on BRENDA, we find that such information is available for not more that 38% of the reactions in different plant models (e.g. 26% of reactions in AraGEM, 38% of reactions in AraCore, but also 27% in the model or rice from Lakshmanan et al. and 21% the models of maize from Seaver et al. and Simons et al). Therefore, this request, like the one about molecular crowding, is infeasible given the current state of plant metabolic modeling. These points are included in the discussion of the updates version of the manuscript.

Based on our additional analyses and thorough consideration of all requests from reviewer #1, we see no additional open points, given both the available data sets as well as the state-of-the-art approaches in constraint-based modeling of plant metabolic networks.

Reviewer #2 (Remarks to the Author):

I appreciate the effort the authors have taken to respond to all of my comments. The manuscript is much clearer now, and I believe all of my questions have been resolved.

We thank the reviewer for the positive evaluation.

Reviewer #3 (Remarks to the Author):

I have only two comments:

(1) I don't think the differences in accuracy (see Fig 3.) are that substantial to justify the title. This is particularly important considering that the difference in accuracy is small and it is based on a single (very small) data set. Will these results replicate in other Arabidopsis data sets. Will these extend to other traits. Will this result hold with larger N and in other species? These are all very important questions that the manuscript cannot answer. For that reason I would suggest a more sober tone in the title (make it more descriptive, e.g. Integrating Metabolic Models into Genomic Selection), abstract, discussion and conclusions.

We thank the reviewer for the insightful comments. Our study was motivated by the lack of understanding of whether or not the performance of classical GS can be improved. We understand that the prediction accuracies are not high (say, above 70%), but this also holds for the classical GS, which is nevertheless employed in practice. To address the reviewer's comments we conducted the following:

- **We modified the title, abstract, and conclusions, as suggested, to tone down the claims.**
- **We stress that netGS is suitable for predictions of traits directly related to metabolism, as stated in the abstract and discussion of the manuscript. In the revised version of the manuscript, we further emphasized that additional research is needed to expand the applicability of netGS to other traits, e.g. yield.**
- **In the revised version of the manuscript, we demonstrated that netGS shows improved performance in another environment, namely <u>low carbon</u> (~10% with genome-wide SNPs and ~50% for enzymatic SNPs).**
- **We emphasize that netGS requires data sets to obtain genotype-specific models, which is the main reason why this proof-of-concept study is conducted in Arabidopsis with the existing data sets.**

(2) I am not 100% satisfied with the answer about the cross-validation. My concern is not with genetic accessions but on weather data from testing set was in any steps of the model building. No data from testing sets should be used for model building, otherwise the estimated accuracies will be upwardly bias. By model building I include anything that may

involve estimation of flux, flux distribution, feature selection, parameter estimation etc. The model must be entirely build within each of the training sets and then just applied by using only the SNP data from the testing set. Please confirm if this was the case.

We stress that data from the testing set <u>were not used in any step</u> of the model building. Therefore, we are confident that the raised issue is addressed.

REVIEWERS' COMMENTS:

Reviewer #1 (Remarks to the Author):

"To the best of our knowledge, such data set is not available neither for rice nor for maize, for which high-quality metabolic models exists (as argued in the main text as well). Developing such a data set would require at least two more years of experiments and measurements, which we find is an unjustified request for a computational systems biology study."
> Computational systems biology study without experimental validation or justification may not be relevant to Nat. Comm. I strongly requested the experimental validation, but if it can't be done, it would be better to submit it to other relevant journal accepting the theoretical study.

"The other point in the first round of reviews dealt with the existence of multiple solutions in the process of determining flux distributions, and the reviewer suggested (as an example) to use unbiased FBA approach. To the best of our knowledge, there is no such FBA approach, so additional analysis in this direction could not be performed. In fact, we went on to analyze the robustness of the flux distributions both in the reference genotype (Col-0) as well as in the flux distributions of the other genotypes—and demonstrated little to no variability in the fluxes. These findings, therefore, demonstrated that our original approach does not suffer from issue of alternative optima."
> It is not acceptable to claim no alternative optima. In the underdetermined linear system, a number of variable (fluxes) is smaller than a number of equations (mass balance in terms of metabolites). I found there is no effort to address the flux variability issue which is actually common topic in the constraint based flux analysis community.

We would like to stress that we do show experimental verification of the approach, since we use data from different environment to demonstrate the prediction accuracy— namely, low nitrogen (in the original and revised version) and low carbon
> I feel that replying on other published works and data may not be enough to validate the results.

Finally, the request to perform FBA with other relevant constraint, like molecular crowding or kinetic information, are simply infeasible given the current state of plant metabolic modeling
> This request is one of my suggestions to improve the quality of the manuscript. If it is infeasible, it may be very hard for me to find any significance or novelty of the current work.

Reviewer #3 (Remarks to the Author):

Judging from the rebuttal letter and the text highlighted text, there have not been major changes in the manuscript.

In particular, regarding the two points that I raised:

(1) Moderating the tone of the title, abstract and discussion, although they change the title the spirit of it remains the same. And I do not see any major moderation of the conclusions in the abstract and discussion. The authors seem to underestimate that (a) the uncertainty about the estimated accuracies is substantial and that (b) this is even more important when considering that the results are based on a small, very peculiar (in terms of selection history and structure) data set. Thus, for me, it remains to be seen whether these advances will be realized when one applies this approach to breeding species that have been subject to a recent history of very intensive selection and for which larger data sets are available to train GS within highly homogeneous populations. I think that the ideas proposed here are novel and that what the manuscript provides is a first evaluation the results of which needs to be taken with caution. Unfortunately, this is not reflected in the manuscript.

(2) The authors state that no aspect of model definition and training was conducted involving data that was not used for validation. This addresses my concern.

Response to reviewers' comments on

‘Integrating molecular markers into metabolic models improves genomic selection for Arabidopsis growth’

Hao Tong, Anika Küken, Zoran Nikoloski

REVIEWERS' COMMENTS:

Reviewer #1 (Remarks to the Author):

“To the best of our knowledge, such data set is not available neither for rice nor for maize, for which high-quality metabolic models exists (as argued in the main text as well). Developing such a data set would require at least two more years of experiments and measurements, which we find is an unjustified request for a computational systems biology study.”

> Computational systems biology study without experimental validation or justification may not be relevant to Nat. Comm. I strongly requested the experimental validation, but if it can’t be done, it would be better to submit it to other relevant journal accepting the theoretical study.

Response: We have tested the application of the proposed netGS approach on data from a low carbon condition, included in the last version of the manuscript. We are currently setting up collaborations to gather the required data set in rice.

“The other point in the first round of reviews dealt with the existence of multiple solutions in the process of determining flux distributions, and the reviewer suggested (as an example) to use unbiased FBA approach. To the best of our knowledge, there is no such FBA approach, so additional analysis in this direction could not be performed. In fact, we went on to analyze the robustness of the flux distributions both in the reference genotype (Col-0) as well as in the flux distributions of the other genotypes—and demonstrated little to no variability in the fluxes. These findings, therefore, demonstrated that our original approach does not suffer from issue of alternative optima.”

> It is not acceptable to claim no alternative optima. In the underdetermined linear system, a number of variable (fluxes) is smaller than a number of equations (mass balance in terms of metabolites). I found there is no effort to address the flux variability issue which is actually common topic in the constraint based flux analysis community.

Response: We do not claim that there are no alternative optima. In contrast, our robustness analysis in fact shows that the accuracy of the predictions from netGS is not affected by the presence of alternative optima, which do investigate with two different approach, initially FBA and in the revised version pFBA. Therefore, our analyses and results are in contrast to what is claimed by the reviewer.

We would like to stress that we do show experimental verification of the approach, since we use data from different environment to demonstrate the prediction accuracy— namely, low nitrogen (in the original and revised version) and low carbon.

> I feel that replying on other published works and data may not be enough to validate the results.

Response: We find that the usage of independent data sets which have not been used in

learning the flux models based on netGS is a well-accepted validation strategy. We do not see how model validation would be affected or would differ if we use a published or newly generated data set for validation.

Finally, the request to perform FBA with other relevant constraint, like molecular crowding or kinetic information, are simply infeasible given the current state of plant metabolic modeling
> This request is one of my suggestions to improve the quality of the manuscript. If it is infeasible, it may be very hard for me to find any significance or novelty of the current work.

Response: As already argued in our response to the previous version of the manuscript, data to impose such constraints are not available in plants, in contrast to unicellular organisms (i.e. *E. coli* and yeast). Therefore, we cannot see how this valuable suggestion can be implemented given the present state-of-the-art in modeling of plant metabolism.

Reviewer #3 (Remarks to the Author):

Judging from the rebuttal letter and the text highlighted text, there have not been major changes in the manuscript. In particular, regarding the two points that I raised:

(1) Moderating the tone of the title, abstract and discussion, although they change the title the spirit of it remains the same. And I do not see any major moderation of the conclusions in the abstract and discussion. The authors seem to underestimate that (a) the uncertainty about the estimated accuracies is substantial and that (b) this is even more important when considering that the results are based on a small, very peculiar (in terms of selection history and structure) data set. Thus, for me, it remains to be seen whether these advances will be realized when one applies this approach to breeding species that have been subject to a recent history of very intensive selection and for which larger data sets are available to train GS within highly homogeneous populations. I think that the ideas proposed here are novel and that what the manuscript provides is a first evaluation the results of which needs to be taken with caution. Unfortunately, this is not reflected in the manuscript.

Response: We thank the reviewer for acknowledging the novelty of the approach, and for raising these points.

We have modified the title to "Integrating molecular markers into metabolic models improves genomic selection for Arabidopsis growth".

In addition, we modified the abstract to explicitly state that the conclusions hold on the data set of 67 accessions used and that the usefulness of the approach in crop research should be determined by generation of matching data sets, as:

"We demonstrate that, comparing to classical genomic selection on the available data sets for 67 accessions, our approach improves the prediction accuracy for growth within and across nitrogen environments by 32.6% and 51.1%, respectively, and from optimal nitrogen to low carbon environment by 50.4%. Therefore, integration of molecular markers into metabolic models offers a novel approach to predict traits directly related to metabolism, and its usefulness in breeding can be examined by gathering matching data sets in crops."

Finally, we added another section to the discussion in which: (a) we comment on the uncertainties on the estimated accuracies and the small data set and population used, (b) we point at factor that can affect the predictions following the proposed approach, and (c) we clearly state further research and data generation are needed to test the performance of the

approach in different crop populations. The following includes the modified sentences and paragraphs:

"We demonstrate that netGS provides the means to integrate molecular markers in large-scale metabolic models, which on the data set from 67 *A. thaliana* accessions resulted in improved prediction of plant growth using enzymatic and genome-wide SNPs."

"Our study provides a proof-of-principle that netGS provides a feasible approach to predict growth for the model plant A. thaliana. The approach is tested by using accession-specific models which integrate metabolomics data from A. thaliana rosettes in respective condition- and accession-specific biomass reactions for the relatively small population. Future simulation studies will examine how changes in the size of the used population may affect the prediction accuracies, which in the examined data sets show comparable uncertainties with those resulting from the classical GS."

"Due to the constraint-based formulation, netGS appears facile to apply for traits directly related to metabolism in agronomically relevant crops. However, testing the performance of netGS on metabolic traits in crops, e.g. maize and rice, that have experienced recent history of intense selection will necessitate the assembly of high-quality metabolic models. Another difficulty is that the models must be able to reproduce growth and other metabolic traits of representative organs or the plant as a whole, before they can be employed for the estimation of fluxes, as intermediate traits. In addition, dedicated experiments will have to be designed to assemble accession-specific biomass reactions which are able to simulate metabolic traits of interest in specific contexts. Therefore, the usage of netGS in decreasing the phenotyping effort and expediting the development of superior crop genotypes remains to be validated in future studies that address the aforementioned challenges."

(2) The authors state that no aspect of model definition and training was conducted involving data that was not used for validation. This addresses my concern.

Response: We thank the reviewer for acknowledging this fact in our modeling study.
